# A framework to mine laser microdissection-based omics data and uncover regulators of pancreatic cancer heterogeneity

**DOI:** 10.1093/gigascience/giaf101

**Published:** 2025-09-05

**Authors:** Pierluigi Di Chiaro, Giuseppe R Diaferia, Gioacchino Natoli, Iros Barozzi

**Affiliations:** Department of Experimental Oncology, IEO, European Institute of Oncology IRCCS, 20139 Milano, Italy; Center for Cancer Research, Comprehensive Cancer Center, Medical University of Vienna, 1090 Vienna, Austria; Department of Experimental Oncology, IEO, European Institute of Oncology IRCCS, 20139 Milano, Italy; Department of Experimental Oncology, IEO, European Institute of Oncology IRCCS, 20139 Milano, Italy; Center for Cancer Research, Comprehensive Cancer Center, Medical University of Vienna, 1090 Vienna, Austria

**Keywords:** bioinformatics, transcriptomics, omics

## Abstract

**Background:**

Pancreatic ductal adenocarcinoma (PDAC), the most common and aggressive form of pancreatic cancer, exhibits profound intratumor morphological heterogeneity, complicating the elucidation of the underlying molecular mechanisms driving its progression.

**Results:**

We present and validate an optimized framework for RNA sequencing (RNA-seq) of multiple spatially resolved laser micro-dissected tumor areas (LMD-seq), along with methodological and analytical details to maximize reproducibility and data mining. This approach enhances sensitivity in detecting lowly expressed genes, outperforming single-cell RNA-seq methods, particularly in identifying rare tumor cell populations and transcriptional programs with low expression. We also present a detailed map of predicted regulatory networks underlying distinct PDAC morpho-biotypes, revealing novel mechanisms and key regulators associated with each subtype.

**Conclusions:**

This study provides fully reproducible workflows, including processed data objects, documented code, and computational predictions of the regulatory activities, enabling robust exploration of intratumor heterogeneity of PDAC. The proposed methodology, datasets, and catalog of the molecular and regulatory mechanisms offer a framework for future studies and applications in PDAC and other cancers.

## Data Description

### Context

Pancreatic ductal adenocarcinoma (PDAC) stands as the most prevalent form of pancreatic cancer and ranks among the most lethal solid malignancies, with an extremely aggressive behavior. Despite advancements over the past 50 years, the 5-year survival rate for patients with PDAC remains critically low, with most patients ineligible for surgery still facing a median life expectancy of less than 6 months [1]. The limited response to chemotherapy of PDAC is largely due to its cellular and architectural heterogeneity, with tumor cells enmeshed in a dense fibrotic stroma that makes up to 90% of the tumor mass. PDACs exhibit varying proportions of tumor cell islands, which are organized either as pseudo-glandular structures or as compact nests of cells with diverse sizes and shapes [2].

This intricate morpho-functional intratumor heterogeneity challenges transcriptomic studies, as standard approaches and bioinformatics pipelines struggle to capture the full spectrum of diversity within the tumor. Bulk RNA sequencing (RNA-seq) studies average transcriptional differences among coexisting cell populations, capturing only the most abundant tumor cells [3]. While single-cell RNA sequencing (scRNA-seq) reveals intratumor heterogeneity at the cellular level, it falls short of pinpointing the exact localization and morphological correlates of these diverse populations [4–6].

Recently, we generated a comprehensive transcriptomic dataset of morphologically distinct tumor regions from patients with PDAC [7]. By employing laser micro-dissection (LMD) coupled with RNA-seq, we profiled multiple tumor areas (each containing 200–500 cells) from treatment-naive patients (an approach we refer to as LMD-seq). This spatially resolved approach enabled the identification of PDAC morpho-biotypes, 3 major coexisting tumor cell states, each characterized by distinct histologic features and transcriptional programs.

**Figure 1:**
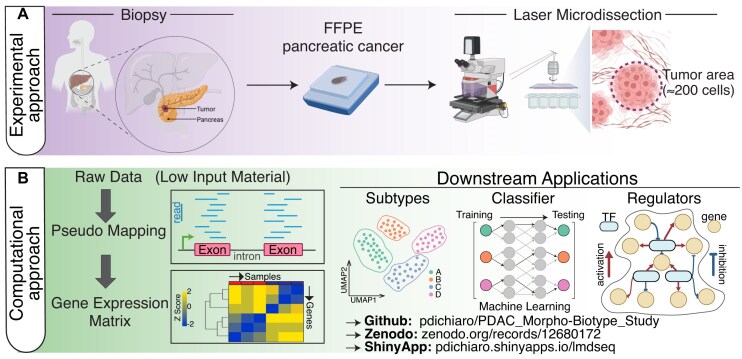
Schematic of the optimized laser micro-dissection approach (LMD-seq). (A, B) Diagram of LMD of small tumor areas (A) and data analysis (B), including preprocessing steps and downstream applications.

In this study, we present an optimized LMD-seq pipeline, together with a detailed description of the computational analyses designed to address the challenges posed by low quantities of fragmented RNA and short sequencing reads from LMD–formalin-fixed, paraffin-embedded (FFPE) samples. In addition to a detailed description of the tools for accessing, reproducing, and reusing the dataset according to Findable, Accessible, Interoperable, Reusable (FAIR) principles [8], we introduce a comprehensive and accessible framework that includes (i) in-depth evaluation of data quality, (ii) pseudo-alignment tools optimized for short sequencing reads to enhance the accuracy of gene expression quantification, (iii) integration with structural genetic variants to assess tumor purity, (iv) comparison with a patient-harmonized single-cell dataset, and (v) advanced computational tools to explore a possible association between different expression programs and their regulators to further explore pancreatic cancer regulatory networks (Fig. 1).

## Methods

### LMD-seq data analysis

#### Read mapping and normalization

Reads were initially preprocessed for quality trimming and adapter clipping using Trim Galore (RRID:SCR_011847) v0.6.5 [9], setting the following parameters: quality = 20, stringency = 3, and length = 20. After the preprocessing steps, the initial 3 nucleotides of the second read, originating from the G-overhang of the template-switching oligonucleotide, were removed. Paired-end reads with a length of at least 20 bp per end were then aligned to the human genome (GRCh38/hg38) using TopHat2 (v2.1.1) [10], STAR (RRID:SCR_004463) v2.7.11 [11], or Kallisto (RRID:SCR_016582) v0.46.0 [12] with default settings and in the correct orientation (–rf-stranded). The GENCODE (RRID:SCR_014966) gene annotations (version 33) were used as the reference transcriptome [13]. Expression counts were then estimated from TopHat2 aligned data using the summarizeOverlaps() function of the GenomicRanges R package (v1.38) [14] with the “IntersectionStrict” mode and list of exons by gene() as features. Transcript-level expression data obtained by Kallisto were imported using the tximport R package (v1.14.2) [15], and the read counts were normalized across samples using the median of ratios method in DEseq2 (v1.26.0) [16]. The normalized counts for protein-coding genes were then log2-transformed and used as a proxy for expression in downstream analyses.

#### Morpho-biotype identification

Tumor morpho-biotype identification was performed as previously described [7]. Briefly, the variance of each gene was calculated using the rowVars() function from the matrixStats R package (v0.59.0) [17] on normalized expression values. Genes were ranked by variance and used for sample clustering with the ConsensusClusterPlus R package (v1.50.0) [18]. Clustering was conducted using Pearson’s correlation distance, the *k*-means algorithm, and 1,000 bootstrap samples. Although *k*-means clustering assumes Euclidean space and spherical clusters, the ConsensusClusterPlus package [18] supports data transformations that enable the use of non-Euclidean distances, including correlation-based measures. These are commonly paired with *k*-means clustering in transcriptomic studies due to their ability to capture expression pattern similarities independently of absolute expression levels and outliers. To mitigate potential biases associated with using a single clustering approach, hierarchical clustering was additionally performed using the hclust() function, with Euclidean distances (after scaling expression values for each gene) computed via the dist() function, and the ward.D2 linkage method from the stats R package (v3.6.2) [19]. Clusters were then determined using the cutreeDynamic() function from dynamicTreeCut R package (v1.63–1) [20] with the following parameters: method = “hybrid,” deepSplit = TRUE, and minClusterSize = 12. The new partition showed high concordance between that obtained by consensus clustering, supporting the robustness of the identified subgroups (Table 1).

**Table 1:** Contingency table summarizing the overlap between clusters derived from hierarchical clustering and morpho-biotype annotations derived from ConsensusClusterPlus

	Cluster					
		1	2	3	4	5
Morpho-biotype	GL	0	21	2	0	8
	TR	0	0	17	0	0
	HY	2	0	0	17	8
	UN	26	0	0	1	0

Each morpho-biotype was then compared against the others in a groupwise manner using the DEseq2 (RRID:SCR_015687) R package (v1.26.0) [16]. Differential expression analysis was performed using the Wald test to compute *P* values for each gene and the Benjamini–Hochberg method [21] to account for multiple hypothesis testing. Genes were deemed significantly differentially expressed if they exhibited an adjusted *P* value ≤0.01 and a linear fold change of at least 2 (either upregulated or downregulated).

#### Copy number variation estimation

Somatic copy number variations (CNVs) for each micro-dissected tumor area were estimated using the inferCNV (RRID:SCR_021140) R package (v1.10.1) [22]. Using normal pancreas samples derived from the Genotype-Tissue Expression project (GTEx) or pseudobulk of endocrine cells derived from scRNA-seq (see below) as reference, inferCNV was run with the following parameters: –cutoff 0.1 and –noise_filter 0.2. To calculate a CNV value quantifying the overall level of CNVs (both duplications and deletions) in each sample, a CNV scoring method [23] was used. CNV scores were re-standardized to a mean of zero, scaled from −1 to 1, and then the sum of squared values was calculated as the overall CNV score for each sample.

#### Enrichment of gene signatures

Transcriptional gene signatures related to biological processes of Synaptic Transmission and Neuronal Differentiation [7] were used to calculate a score value for each sample. AddModuleScore() function of Seurat (RRID:SCR_007322) R package (v5.0.2) [24] was used with standard parameters except for the number of control features, which was set to the number of genes in the signature when possible. Gene set variation analysis (GSVA) and single-sample gene set enrichment analysis (ssGSEA) were also run using the GSVA (RRID:SCR_021058) R package (v2.2.0) [25], with default parameters.

#### Motif enrichment analysis

Considering the sequences from −500 bp to +50 bp relative to annotated transcription start sites of differentially expressed genes of the 3 morpho-biotypes (NCBI Refseq release 200 gene annotations), motif enrichment analysis was performed using Pscan [26] and a custom set of position-specific weight matrices (PWMs) [27]. PWMs were considered significantly overrepresented when showing a *P* value ≤1e-5.

### Analysis of regulatory networks

ARACNe-AP [28] was run with 200 bootstrap iterations for the reconstruction of gene regulatory networks using the gene expression matrix based on LMD data. To identify the relationships among putative regulators and predicted target genes, we used a published list of transcription factors [29] that was further manually curated removing well-known chromatin remodelers and mitochondrial transcriptional regulators (final number of transcription factors [TFs] considered was 1,590). ARACNe networks were imported and used by the VIPER (Virtual Inference of Protein Activity by Enriched Regulon analysis) R package (v1.32.0) [30] for the identification of possible master regulators. We compared each morpho-biotype against each other using the msviper algorithm of the VIPER package and the gene signatures previously identified. To infer the enrichment of the top differentially expressed transcription factors, we measured the activity of a regulator based on the enrichment of its target genes in each morpho-biotype. Representative differentially expressed transcription factors and their gene targets were displayed as networks using Cytoscape (v.3.8.2) [31].

### Enrichment of genomic intervals

To efficiently query and compare genomic interval datasets, Giggle, a search engine for large-scale integrated genome analysis tool (v0.6.3) [32], was used. Promoter regions of target genes identified by the ARACNe-VIPER approach for the TFs FOXA2, HNF1B, MYRF, ZEB1, and TP63 were used as input genomic regions, as previously described. These regions were derived as described above. Significant peaks (*q* < 1E-05, in BED format) from chromatin immunoprecipitation sequencing (ChIP-seq) datasets corresponding to each of these TFs in pancreatic cancer cell lines were downloaded from ChIP-ATLAS (https://chip-atlas.org). Giggle was first used to create an index from these ChIP-seq annotations. We then searched the input promoter regions against this index and assessed the enrichments.

### Single-cell RNA-seq data analysis

#### Dataset processing

Single-cell RNA-seq data from primary treatment-naive PDACs were obtained from Chan-Seng-Yue et al. [5]. Single-cell data (h5 files) already aligned to hg19 using CellRanger (RRID:SCR_023221) v.2.1.1 (10x Genomics) were downloaded from the EGA archive (EGAC00001000710) and then were imported using the Seurat R toolkit [24]. After the removal of low-quality cells (<1,000 detected genes or >25% reads mapping to mitochondrial genome) and of mitochondrial genes from the features, the SCTransform algorithm (v0.4.1) was used to normalize the data [33]. Principal component analysis (PCA) was performed and the top principal components (PCs) (determined as the number of PCs whose percentage of variance increase among consecutive PCs is less than 0.1%) were then used to build a *k*-nearest neighbors graph (*k* = 20) of single-cell profiles. Community detection using the Leiden clustering method (resolution = 0.8) was performed to identify distinct cell clusters, and then different cell populations were identified using known cell type–specific gene markers from [34, 35]. To confirm the presence of clusters enriched for tumor cells, InferCNV (R package; v1.10.1) [22] was used as described above, setting a set of high-confidence nonneoplastic cells identified in the same data.

Although this dataset was aligned to a different reference genome (hg19) than LMD-seq, all downstream analyses—including enrichment score calculations—were performed independently, without direct integration of expression values or annotations between genome builds. As a result, the difference in reference genome did not impact the robustness and interpretability of the downstream results.

#### Dataset Integration of scRNA-seq data

Harmony (RRID:SCR_022206) (R package; v0.1.1) [36] was used to correct for technical factors between datasets. Using patient ID as a factor, Harmony constructed a well-integrated embedding across cell types and patients. After selecting only tumor cells, batch-corrected harmony PCA components were then used as input for the Uniform Manifold Approximation and Projection (UMAP) and a downstream clustering approach. A *k*-nearest neighbors graph (*k* = 20) of single tumor cell profiles was built, and then a Leiden clustering method (resolution = 0.3) was performed to identify distinct tumor cell clusters. Transcriptional signature scores computed by Seurat’s AddModulescore() function as described above (see Enrichment of gene signatures) was displayed using Seurat’s FeaturePlot() function. To generate a pseudobulk of specific cell types across patients, the AggregateExpression() function of the Seurat R package (v5.0.2) [24] was used.

### Quality control

The LMD-seq dataset was previously generated [7] from 30 chemotherapy-naive patients with PDAC (Supplementary Table S1), accurately micro-dissecting a total of 102 tumor samples.

To ensure a high morphological quality from archival FFPE human samples, we optimized our RNA-seq pipeline for micro-dissected tumor areas containing approximately 200 to 500 cells. The sequencing analysis from such samples is inherently challenging due to the tissue processing, which can compromise RNA integrity. The fragmented mRNA can affect gene detection and introduce sequencing artifacts [37]. To mitigate these issues, one of the key optimization steps involved the alignment and counting of sequencing reads on the annotated exons of each mRNA. Initially, we used standard aligners such as TopHat2 and STAR, which rely on genome mapping to identify sequencing positions in the genome potentially originating the read. However, this method showed low performance in quantifying their abundances, with only about 2% to 5% of mapped reads on exons and 1.4% to 2.4% of unique reads being quantified accurately. Among the two, STAR performed slightly better than TopHat2 (Supplementary Table S2). Conversely, Kallisto’s pseudomapping approach, which employs a statistical framework to account for all the potential transcripts of origin for each sequencing read, significantly enhanced both the accuracy and reliability of gene expression quantification. This method mapped approximately 64% to 76% of the reads on exons and more than doubled the final number of unique reads, achieving 3.6% to 4.7%. On average, pseudomapping recovered approximately 3.4 million reads per sample, compared to approximately 1.6 million per sample using traditional aligners (Supplementary Table S2). Thus, by employing pseudomapping instead of traditional genome mapping, we achieved higher read recovery, enhancing the sensitivity of fragmented RNA quantification from human FFPE laser micro-dissected samples.

The analysis of 102 micro-dissected tumor areas, generated by LMD-seq, unveiled 4 distinct PDAC cell states (or morpho-biotypes)—glandular (GL), transitional (TR), hybrid (HY), and undifferentiated (UN)—each characterized by distinct morphological profiles and transcriptional properties [7].

**Figure 2:**
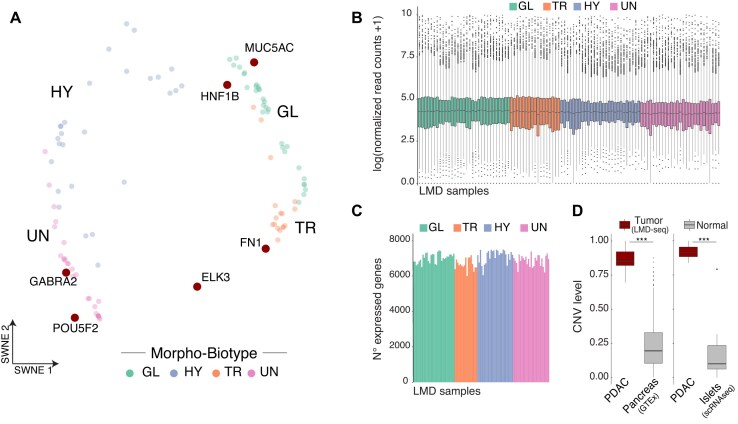
LMD-seq generates high-depth, high-quality transcriptomic data. (A) SWNE representation of single tumor areas from LMD-seq, colored by morpho-biotype. Co-embedded genes (2 for each morpho-biotype) are highlighted (dark red). (B) Box plot showing the coverage distribution for all micro-dissected samples, grouped by morpho-biotype. (C) Bar plot showing the number of detected genes for all micro-dissected samples, grouped by morpho-biotype. (D) Box plot showing the CNV score values calculated in tumor PDAC samples (LMD-seq) compared with normal pancreas samples (GTEx) or pseudobulk of pancreatic endocrine islets (scRNA-seq). *P* value calculated using 2-sided Wilcoxon rank-sum tests. GL, *n* = 31; TR, *n* = 17; HY, *n* = 27; UN, *n* = 27.

We applied the similarity weighted nonnegative embedding (SWNE) approach to visually confirm a clear separation of morpho-biotypes in a dimensionally reduced space and to display the key genes contributing to each group (Fig. 2A).

The technical quality of LMD-seq was evaluated by examining the coverage distribution for all samples, which determined high-quality data distribution across the morpho-biotype annotation for each sample. This result ruled out the possibility of gene expression changes due to technical biases (Fig. 2B). To verify the robustness of the data, we calculated the number of detected genes (genes with at least 1 normalized read count per kb) for each sample across morpho-biotypes. The results were consistent with those obtained from canonical bulk RNA-seq (Fig. 2C).

The estimate of CNV levels was calculated for each sample, combining duplications and deletions. The micro-dissected samples showed higher CNV levels, compared to either bulk, normal pancreatic tissue from GTEx, or a pseudobulk of normal endocrine cells derived from scRNA-seq data (Fig. 2D). This supports the precise dissection of areas highly enriched in tumor cells, with minimal contamination by noncancer cells during the micro-dissection procedure.

Taken together, these analyses support the high-quality of the generated transcriptomic profiles (Supplementary Table S2).

## Data Validation

**Figure 3:**
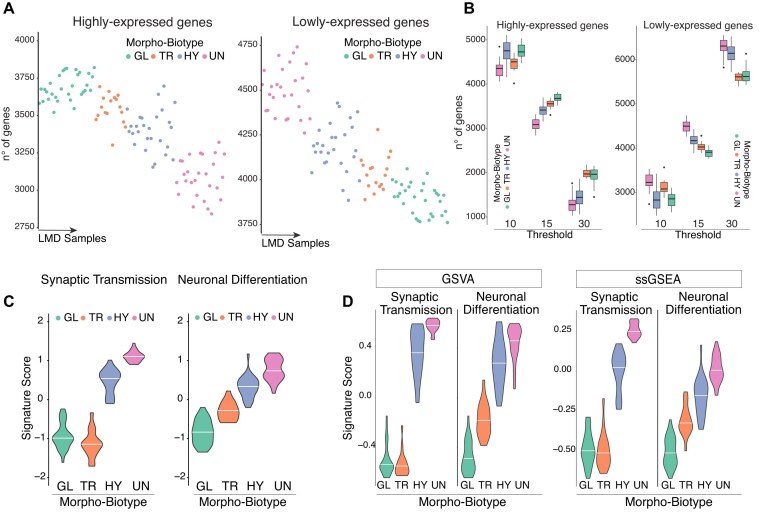
LMD-seq captures genes with low levels of expression. (A) Distributions of the number of highly (left) or lowly expressed (right) genes across LMD tumor samples, grouped by morpho-biotype. The threshold used to distinguish between high- and low-expressed genes was set at 15 normalized gene counts per kilobase. (B) Box plots showing the distributions of the number of genes expressed at a high (left) or low level (right) at different thresholds (10, 15, and 30 normalized gene counts per kilobase). (C, D) Violin plots showing the enrichment of the indicated biological processes (Synaptic Transmission and Neuronal Differentiation), stratified by morpho-biotype based on LMD-seq data, using either Seurat’s AddModuleScore function (C) or GSVA and ssGSEA (D).

Next, we examined the number of expressed genes across different morpho-biotypes. We specifically focused on highly expressed genes (normalized gene counts per kilobase >15) and low-expressed genes (normalized gene counts per kilobase <15) and subsequently calculated the number of expressed genes for each sample (Fig. 3A). Consistent with the results of the study reporting the main findings related to these datasets [7], tumor areas belonging to the undifferentiated morpho-biotype exhibited a lower number of highly expressed genes and a higher number of lowly expressed genes compared to other samples. The results were highly robust across 3 different thresholds, with the undifferentiated morpho-biotype consistently showing fewer highly expressed genes and a greater number of lowly expressed genes relative to other morpho-biotypes (Fig. 3B).

The undifferentiated morpho-biotype resembles progenitor-like cells undergoing neuronal lineage priming, expressing low levels of neuronal differentiation and synaptic transmission processes [7]. Enrichment analysis confirmed that these signatures were significantly associated only in this morpho-biotype (Fig. 3C and Supplementary Table S3). Alternative methods to score the gene signatures, including GSVA and ssGSEA, yielded consistent results (Fig. 3D), supporting high robustness of the results, irrespective of the scoring method chosen.

**Figure 4:**
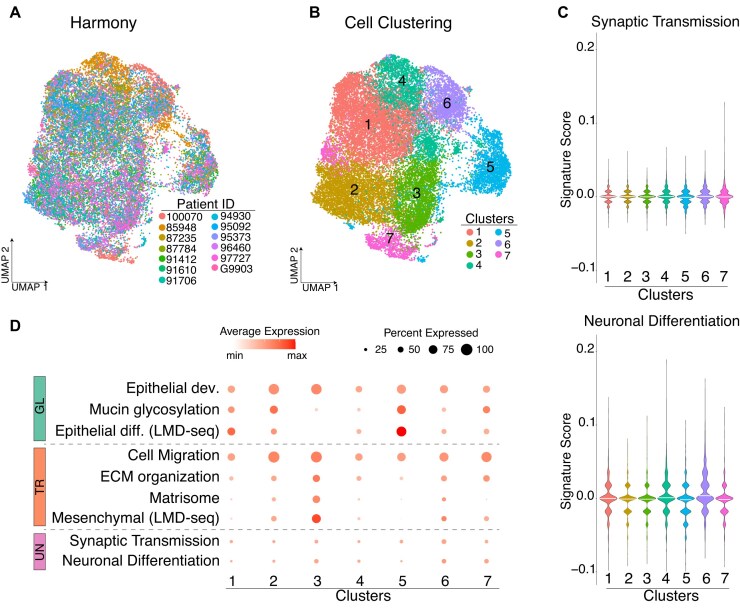
scRNA-seq does not detect the low-expressing neuronal genes of the undifferentiated morpho-biotype. (A, B) UMAP of single tumor cells from scRNA-seq data in 13 primary PDACs after dataset integration using Harmony colored by patient ID (A) or by cell clusters (B). (C) Violin plots showing the enrichment of the indicated biological processes (synaptic transmission and neuronal differentiation), stratified by cell clusters based on scRNA-seq data. (D) Bubble plot showing the enrichment of the indicated gene signatures (“epithelium development,” GOBP; “glycosylation of mucins,” Reactome; “cell migration,” GOBP; positive regulation of extracellular matrix organization, GOBP; PDAC matrisome signature [39]; epithelial differentiation, LMD-seq; mesenchymal, LMD-seq; synaptic transmission, LMD-seq; neuronal differentiation, LMD-seq), across cell clusters. Size of the dot represents the percentage of cells expressing the program, with the color indicating the average gene expression.

Additionally, we compared the performance of our LMD-seq with standard scRNA-seq in detecting low-expressing genes. To achieve this, we employed publicly available scRNA-seq data [5]. After integrating the high-quality CNV-validated single tumor cells from several patients (Fig. 4A), we performed cell clustering and computed the enrichment scores for neuronal differentiation and synaptic transmission processes across different cell clusters (Fig. 4B–D and Supplementary Table S3). The scRNA-seq data were not able to discriminate tumor cells enriched in nontypically endodermal pathways, such as neuronal differentiation and synaptic transmission. This confirms the superior capability of our LMD-seq approach compared to the commonly used scRNA-seq [7] in identifying rare tumor cells and capturing low-expressed genes. As further validation of these negative results and generally of our approach, LMD-seq was able to identify enrichment in specific cell clusters for canonical gene programs consistent with the morpho-biotype programs identified through our LMD-seq analysis. Biological processes, such as epithelial development and mucin glycosylation, were enriched in clusters corresponding to epithelial differentiation, typical of the glandular morpho-biotype. In contrast, processes related to cell migration and extracellular matrix (ECM) organization were enriched in the transitional morpho-biotype, in line with its mesenchymal-like expression profile. Notably, a matrisome gene signature [38] was also enriched in clusters corresponding to the transitional morpho-biotype, further confirming the important role of ECM remodeling in the tumor microenvironment (Fig. 4D).

### Reuse potential

**Figure 5:**
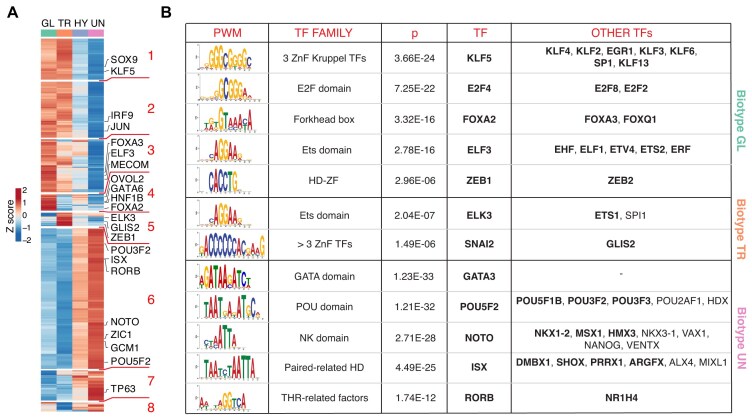
Transcription factors predicted to regulate the morpho-biotypes. (A) Heatmap showing TFs differentially expressed in PDAC morpho-biotypes. Highly differential TFs (Fold Change ≥ 3) and selected relevant TFs are shown. Gene expression modules (#1–8), derived from differentially expressed genes clustered by graph-based clustering approach, are indicated in red on the right. Morpho-biotype annotations are shown (GL, TR, HY, UN). (B) Motif enrichment analysis at the promoters of differentially expressed genes (from −500 to +50 bp relative to the annotated transcription start site). The table shows the PWMs overrepresented in 1 morpho-biotype relative to the others (*P* ≤ 1E-5), their TF family, and their cognate TFs. TFs identified as differentially expressed in different morpho-biotypes are labeled in bold.

Finally, we aimed to identify the transcriptional regulatory networks that sustain the different PDAC morpho-biotypes as one of the potential downstream applications of our dataset. A significant number of TFs were differentially expressed in each morpho-biotype (Fig. 5A). These included known endodermal lineage TFs in the glandular morpho-biotype (e.g., SOX9, KLF5, HNF1B, GATA6, ELF3, and FOXA2) [27, 39–42], EMT-associated TFs in the transitional morpho-biotype (e.g., ZEB1 and GLIS2) [43], and TFs related to neural development (POU5F2, POU3F2, NOTO), as well as stemness of basal epithelia (TP63) in the undifferentiated morpho-biotype.

**Figure 6:**
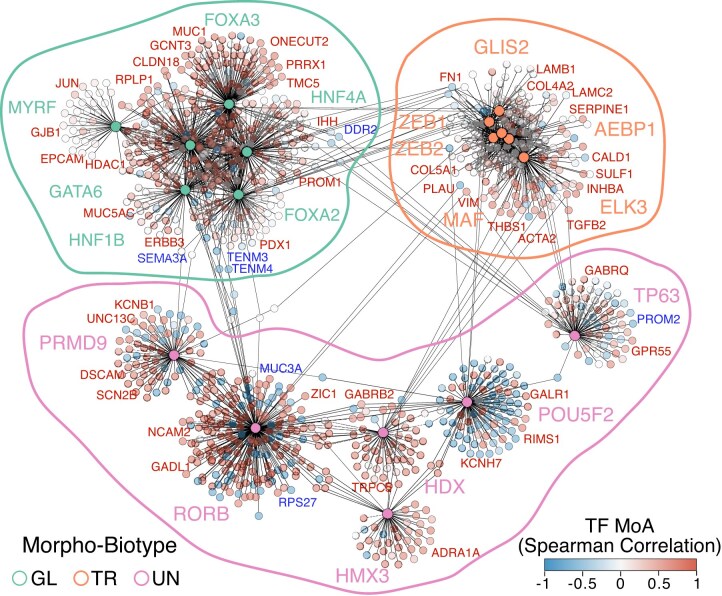
Morpho-biotype-specific transcriptional regulatory networks. Gene regulatory network representation of selected, differentially activated TFs in the 3 indicated morpho-biotypes, as determined by ARACNe-VIPER [28, 30]. Each node represents a target gene that is predicted to be positively (red) or negatively (blue) regulated by its corresponding TF (TF MoA, mode of action of a TF based on Spearman’s correlation coefficient with expression of its target genes).

To determine their potential roles in establishing morpho-biotype specific regulatory networks, we first analyzed the statistical overrepresentation of TF DNA binding motifs in the promoters of differentially expressed genes characteristic of each morpho-biotype (Fig. 5B and Supplementary Table S4). We confirmed the presence of motifs for endodermal lineage TFs such as KLF5, ELF3, and FOXA as enriched in the promoters of genes overexpressed in the glandular morpho-biotype. In addition, motifs bound by ZEB1 were enriched in these promoters. ZEB1 is well known for acting as a transcriptional repressor of epithelial genes [43]. Although we predict it to be more active in the transitional subtype (Fig. 6 and Supplementary Table S4), its binding motif is enriched in promoters of genes typically expressed in the glandular subtype, suggesting ZEB1-mediated repression of epithelial genes in transitional-type cells.

Binding motifs for the EMT activator SNAI2 [44] were instead enriched in the transitional group. On the other hand, promoters of genes overexpressed in the undifferentiated morpho-biotype were enriched for motifs bound by several neuronal lineage TFs such as POU5F2 and other family members, as well as the homeobox TFs NOTO, NKX1-2, and HMX3.

In addition, we sought to identify the transcriptional regulatory networks linking TFs to their target genes across different morpho-biotypes. To achieve this, we first employed a reverse engineering approach to reconstruct the regulatory networks from LMD-seq data, identifying relationships between putative regulators and predicted target genes using ARACNe-AP [28]. This tool quantifies the statistical dependence between expression levels by analyzing shared expression patterns across multiple samples, generating a gene–pair association matrix. This matrix is then imported into the VIPER algorithm [30] to identify the most active TFs based on the enrichment of their predicted target genes in each morpho-biotype (Fig. 6A and Supplementary Table S4). This analysis not only linked TFs associated with endodermal lineage or EMT programs to their activated and repressed targets but also confirmed the presence of an active neuronal-like network in the undifferentiated morpho-biotype. It suggested possible direct links between neural-enriched TFs and genes associated with functional categories characteristic of the nervous system.

To further validate our findings, we assessed the enrichment in the promoter regions of predicted target genes within each morpho-biotype-specific regulon, for the binding of the corresponding regulators (as assessed by publicly available ChIP-seq obtained from pancreatic cancer cells). Despite the analysis being restricted to promoter regions, which represent only a small subset of the regulatory genome, we observed a statistically significant overlap across all regulons analyzed (Giggle [32]; *P* < 1E-2, Fisher’s exact test; Supplementary Table S4). This independent approach provides additional support for the validity of the regulatory associations identified using VIPER.

In summary, our data provide a comprehensive reference and resource for understanding the molecular and regulatory mechanisms driving PDAC heterogeneity, highlighted by the identification of novel morpho-biotypes [7]. We further demonstrated that LMD-seq is a valuable spatial transcriptomics approach, capable of robustly capturing histological traits and gene expression patterns. This workflow achieved unprecedented specificity and sensitivity, using as few as ~200 cells, combined with optimized computational analysis. It offers a cost-effective method for conducting discovery studies in several histologically relevant models. To support the exploration and reuse of the LMD-seq dataset, we deployed an interactive data visualization platform [45], enabling researchers and clinicians to intuitively navigate transcriptional profiles of laser micro-dissected PDAC samples, considering individual samples, morpho-biotypes, and genes. This web-based tool enhances transparency, promotes data discovery, and simplifies the integration of LMD-seq datasets into diverse research workflows.

## Availability of Source Code and Requirements

- Project name: PDAC Morpho-Biotypes
- Project homepage: https://github.com/pdichiaro/PDAC_Morpho-Biotype_Study.
- Operating system(s): Linux operating system
- Programming language: Bash, R
- Other requirements: Anaconda
- License: GNU GPL

The LMD-seq scripts, including processing pipeline, from read trimming to pseudo-mapping, are available online on GitHub [46], with the corresponding files available on Zenodo [47].

Data analysis R scripts for data visualization and further downstream analysis are also available online on GitHub [46], with the corresponding files available on Zenodo [47]. An archival copy of the code is available via Software Heritage [48].

## Supplementary Material

giaf101_Supplemental_Files

giaf101_Authors_Response_To_Reviewer_Comments_original_Submission

giaf101_Authors_Response_To_Reviewer_Comments_Revision_1

giaf101_Authors_Response_To_Reviewer_Comments_Revision_2

giaf101_GIGA-D-24-00581_Original_Submission

giaf101_GIGA-D-24-00581_Revision_1

giaf101_GIGA-D-24-00581_Revision_2

giaf101_GIGA-D-24-00581_Revision_3

giaf101_Reviewer_1_Report_Original_Submission Thazin Aung -- 2/2/2025

giaf101_Reviewer_1_Report_Revision_1 Thazin Aung -- 6/10/2025

giaf101_Reviewer_1_Report_Revision_2 Thazin Aung -- 7/22/2025

giaf101_Reviewer_2_Report_Original_Submission Qingnan Liang, Ph.D. -- 3/21/2025

## Data Availability

The LMD-seq data, including sequencing raw data, were deposited in the Gene Expression Omnibus (GEO) database, accession number GSE209952. The scRNA-seq data used in this study are available via the European Genome-Phenome Archive (EGA), accession number EGAD00010001811 [45]. The annotated LMD-seq dataset can be accessed and browsed via an interactive Shiny app [49], providing researchers with a user-friendly platform to explore the data also without any background in computational biology.

## Additional Files

**Supplementary Table S1**. Clinical features of the patient cohort included in the study.

**Supplementary Table S2**. Summary of RNA sequencing alignment results. The table provides the following information: (a) comparisons of sequencing results of 10 LMD-seq PDAC samples aligned using TopHat2, STAR, or Kallisto and (b) sequencing results of all LMD PDAC samples aligned using Kallisto. Sample annotations and number of expressed genes for each sample are also reported.

**Supplementary Table S3**. Gene signatures of synaptic transmission and neuronal differentiation. The table provides the following information: (a) the list of expressed genes annotated to GO terms related to synaptic transmission processes, (b) the list of expressed genes annotated to GO terms related to neuronal differentiation processes, (c) the manually curated list of expressed genes related to neuronal differentiation processes, and (d) a detailed description of the gene signature construction.

**Supplementary Table S4**. Gene regulatory networks in distinct morpho-biotypes. The table provides the following information: (a) the list of all differentially expressed transcription factor genes, (b) the position weight matrices (PWMs) used in the motif enrichment analyses, and (c) the master regulators derived by the VIPER analysis in the different morpho-biotypes. For each PWM, the source (database or publication) is indicated, while for each master regulator, the gene expression levels and log2FC are shown. (d) Results of the enrichment analysis derived by the Giggle tool for some morpho-biotype-specific regulators. For each regulon, the corresponding ChIP-seq dataset and its annotation are indicated. The table also reports the odds ratio, estimating the enrichment of observed versus expected overlaps; Fisher’s exact test statistics and associated *P* values; the combined score, which integrates enrichment magnitude and significance; and the percentage of target genes within the regulon that show evidence of binding in the ChIP-seq dataset.

## Abbreviations

CDF, cumulative distribution function; ChIP-seq: chromatin immunoprecipitation sequencing; CNV, copy number variation; ECM: extracellular matrix; FAIR, Findable, Accessible, Interoperable, Reusable; FFPE, formalin-fixed, paraffin-embedded; GL, glandular; GSVA, gene set variation analysis; GTEx, Genotype-Tissue Expression project; HY, hybrid; LMD, laser micro-dissection; NCBI, National Center for Biotechnology Information; PC: principal component; PCA, principal component analysis; PDAC, pancreatic ductal adenocarcinoma; PWMs, position-specific weight matrices; RNA-seq, RNA sequencing; scRNA-seq, single-cell RNA sequencing; ssGSEA, single-sample gene set enrichment analysis; SWNE: similarity weighted nonnegative embedding; TF, transcription factor; TR, transitional; UMAP, Uniform Manifold Approximation and Projection; UN, undifferentiated.
