# Supplementary material for: A framework to mine laser microdissection-based omics data and uncover regulators of pancreatic cancer heterogeneity
Source: Gigascience. 2025 Sep 5;14:giaf101. doi: 10.1093/gigascience/giaf101 (PMC12412123; doi:10.1093/gigascience/giaf101)
Supplement: giaf101_GIGA-D-24-00581_Original_Submission [file giaf101_giga-d-24-00581_original_submission.pdf]

GigaScience

A framework to mine laser microdissection-based omics data and uncover regulators of pancreatic cancer heterogeneity

--Manuscript Draft--

**Manuscript Number:**	GIGA-D-24-00581
**Full Title:**	A framework to mine laser microdissection-based omics data and uncover regulators of pancreatic cancer heterogeneity
**Article Type:**	Data Note

Funding Information:

Fondazione AIRC per la ricerca sul cancro ETS (fellowship for Italy)	Dr. Pierluigi Di Chiaro
Medizinische Universität Wien	Assist. Prof. Iros Barozzi
Ministero della Salute (GR-2016-02361721)	Dr. Giuseppe R. Diaferia
Fondazione AIRC per la ricerca sul cancro ETS (27555)	Dr. Gioacchino Natoli
Fondazione AIRC per la ricerca sul cancro ETS (21147)	Dr. Gioacchino Natoli
Ministero della Salute - ricerca corrente (IEO IRCCS)	Not applicable
Ministero della Salute - 5x1000 (IEO IRCCS)	Not applicable
Ministero della Salute - ricerca corrente (alleanza contro il cancro)	Not applicable

Abstract:

Background

Pancreatic ductal adenocarcinoma (PDAC), the most common and aggressive form of pancreatic cancer, exhibits profound intra-tumour morphological heterogeneity, complicating the elucidation of the underlying molecular mechanisms driving its progression.

Results

We present and validate an optimized framework for RNA sequencing of multiple spatially resolved laser micro-dissected tumor areas (LMD-seq), along with methodological and analytical details to maximize reproducibility and data mining. This approach enhances sensitivity in detecting lowly-expressed genes, outperforming single-cell RNA-seq methods, particularly in identifying rare tumor cell populations and transcriptional programs with low expression. We also present a detailed map of predicted regulatory networks underlying distinct PDAC morpho-biotypes, revealing novel mechanisms and key regulators associated with each subtype.

Conclusions

This study provides fully reproducible workflows, including processed data objects, documented code, and computational predictions of the regulatory activities, enabling robust exploration of intra-tumor heterogeneity of PDAC. The proposed methodology, datasets, and catalogue of the molecular and regulatory mechanisms offer a framework for future studies and applications in PDAC and other cancers.

**Corresponding Author:**	Iros Barozzi, PhD<br>Medical University of Vienna: Medizinische Universitat Wien<br>Vienna, Select One AUSTRIA
**Corresponding Author Secondary Information:**	
**Corresponding Author's Institution:**	Medical University of Vienna: Medizinische Universitat Wien

Powered by Editorial Manager® and ProduXion Manager® from Aries Systems Corporation

Corresponding Author's Secondary Institution:	
First Author:	Pierluigi Di Chiaro
First Author Secondary Information:	
Order of Authors:	Pierluigi Di Chiaro
	Giuseppe R. Diaferia
	Gioacchino Natoli
	Iros Barozzi, PhD
Order of Authors Secondary Information:	
Additional Information:	
Question	Response
Are you submitting this manuscript to a special series or article collection?	No
**Experimental design and statistics**<br><br>Full details of the experimental design and statistical methods used should be given in the Methods section, as detailed in our Minimum Standards Reporting Checklist. Information essential to interpreting the data presented should be made available in the figure legends.<br><br>Have you included all the information requested in your manuscript?	Yes
**Resources**<br><br>A description of all resources used, including antibodies, cell lines, animals and software tools, with enough information to allow them to be uniquely identified, should be included in the Methods section. Authors are strongly encouraged to cite Research Resource Identifiers (RRIDs) for antibodies, model organisms and tools, where possible.<br><br>Have you included the information requested as detailed in our Minimum Standards Reporting Checklist?	Yes
**Availability of data and materials**	Yes

Powered by Editorial Manager® and ProduXion Manager® from Aries Systems Corporation

All datasets and code on which the conclusions of the paper rely must be either included in your submission or deposited in publicly available repositories (where available and ethically appropriate), referencing such data using a unique identifier in the references and in the “Availability of Data and Materials” section of your manuscript.<br><br>Have you have met the above requirement as detailed in our Minimum Standards Reporting Checklist?	
GigaScience has policies and guidelines in place for the use of generative AI-writing tools such as ChatGPT. If you have used such writing tools to assist with writing the manuscript this must be declared and cited in the text. Authors should not list AI-writing tools and other AI-assisted technologies as an author or co-author and should acknowledge that they are fully responsible for text generated or refined by AI-writing tools.<p><br><br>A summary of use (particularly in the introduction or among methods) needs to be included at the end of the paper, and the outputs should also be included as a supplementary file hosted in GigaDB or other open repositories. Please read our guidelines for more information. <p><br><br>By submitting to GigaScience, you are aware of the journal's AI-writing tools policy, and if you have declared use of such tools below, you have acknowledged this where appropriate in your manuscript and have made a summary of use and outputs available. <p><br><b>AI-assisted writing tools have been used in the preparation of this manuscript?	No

Powered by Editorial Manager® and ProduXion Manager® from Aries Systems Corporation

Main Text
Click here to access/download;Manuscript;Dichiaro_ms_GigaScience-Datanote_modified.docx

A framework to mine laser microdissection-based omics data and uncover regulators of pancreatic cancer heterogeneity

Authors
Pierluigi Di Chiaro[1,2], Giuseppe R. Diaferia[1], Gioacchino Natoli[1], Iros Barozzi[2]

Affiliations
1. Department of Experimental Oncology, IEO, European Institute of Oncology IRCCS, Milano, Italy
2. Center for Cancer Research, Comprehensive Cancer Center, Medical University of Vienna, Vienna, Austria

corresponding author(s): Iros Barozzi (iros.barozzi@meduniwien.ac.at)
Pierluigi Di Chiaro (pierluigi.dichiaro@ieo.it)

Abstract

Background
Pancreatic ductal adenocarcinoma (PDAC), the most common and aggressive form of pancreatic cancer, exhibits profound intra-tumour morphological heterogeneity, complicating the elucidation of the underlying molecular mechanisms driving its progression.

Results
We present and validate an optimized framework for RNA sequencing of multiple spatially resolved laser micro-dissected tumor areas (LMD-seq), along with methodological and analytical details to maximize reproducibility and data mining. This approach enhances sensitivity in detecting lowly-expressed genes, outperforming single-cell RNA-seq methods, particularly in identifying rare tumor cell populations and transcriptional programs with low expression. We also present a detailed map of predicted regulatory networks underlying distinct PDAC morpho-biotypes, revealing novel mechanisms and key regulators associated with each subtype.

Conclusions
This study provides fully reproducible workflows, including processed data objects, documented code, and computational predictions of the regulatory activities, enabling robust exploration of intra-tumor heterogeneity of PDAC. The proposed methodology, datasets, and catalogue of the molecular and regulatory mechanisms offer a framework for future studies and applications in PDAC and other cancers.

Keywords
Bioinformatics, Transcriptomics, Omics

Data Description

Context

Pancreatic ductal adenocarcinoma (PDAC) stands as the most prevalent form of pancreatic cancer and ranks among the most lethal solid malignancies with an extremely aggressive behaviour. Despite advancements over the past 50 years, the 5-year survival rate for PDAC patients remains critically low, with most patients ineligible for surgery still facing a median life expectancy of less than 6 months [1]. The limited response to chemotherapy of PDAC is largely due to its cellular and architectural heterogeneity, with tumour cells enmeshed in a dense fibrotic stroma that makes up to 90% of the tumour mass. PDACs exhibit varying proportions of tumour cell islands, which are organized either as pseudo-glandular structures or as compact nests of cells with diverse sizes and shapes [2].

This intricate morpho-functional intra-tumour heterogeneity challenges transcriptomic studies, as standard approaches and bioinformatics pipelines struggle to capture the full spectrum of diversity within the tumour. Bulk RNA sequencing (RNA-seq) studies average transcriptional differences among coexisting cell populations, capturing only the most abundant tumour cells [3]. While scRNA-seq reveals intra-tumour heterogeneity at the cellular level, it falls short of pinpointing the exact localization and morphological correlates of these diverse populations [4-6].

Recently, we generated a comprehensive transcriptomic dataset of morphologically distinct tumour regions from PDAC patients [7]. By employing laser micro-dissection (LMD) coupled with RNA sequencing (RNA-seq), we profiled multiple tumour areas (each containing 200-500 cells) from treatment-naïve patients (an approach we refer to as LMD-seq). This spatially-resolved approach enabled the identification of PDAC morpho-biotypes, three major coexisting tumour cell states, each characterized by distinct histological features and transcriptional programs.

In this study, we present an optimized LMD-seq pipeline, together with a detailed description of the computational analyses designed to address the challenges posed by low quantities of fragmented RNA and short sequencing reads from LMD-FFPE (Formalin-Fixed Paraffin-Embedded) samples. In addition to a detailed description of the tools for accessing, reproducing, and reusing the dataset according to FAIR principles [8], we introduce a comprehensive and accessible framework that includes: *i*) in-depth evaluation of data quality, *ii*) pseudo-alignment tools optimized for short sequencing reads to enhance the accuracy of gene expression quantification, *iii*) integration with structural genetic variants to assess tumour purity, *iv*) comparison with a patient-harmonized single-cell dataset and *v*) advanced computational tools to explore possible association between different expression programs and their regulators to further explore pancreatic cancer regulatory networks (**Figure 1**).

Methods

LMD-seq data analysis

Read mapping and normalization

Reads were initially pre-processed for quality trimming and adapter clipping using Trim Galore (v0.6.5) [9] setting the following parameters: quality=20, stringency=3, and length=20. After the pre-processing steps, the initial three nucleotides of the second read, originating from the G-overhang of the template-switching oligonucleotide, were removed. Paired-end reads with a length of at least 20 base pairs per end were then aligned to the human genome (GRCh38/hg38) using TopHat2 (v2.1.1) [10] or Kallisto (v0.46.0) [11] with default settings and in the correct orientation (--rf-stranded). The GENCODE gene annotations (version 33) was used as the reference transcriptome [12]. Expression counts were then estimated from TopHat2 aligned data using summarizeOverlaps() function of GenomicRanges R package (v1.38) [13] with the "IntersectionStrict" mode and list of exons by gene () as features. Transcript-level expression data obtained by Kallisto was imported using the tximport R package (v1.14.2) [14] and the read counts were normalized across samples using the median of ratios method in DEseq2 (v1.26.0) [15]. The normalized counts for protein-coding genes were then log2-transformed and used as a proxy for expression in downstream analyses.

Morpho-biotype identification

Tumor morpho-biotype identification was performed as previously described in [7]. Briefly, the variance of each gene was calculated using the rowVars() function from the matrixStats R package (v0.59.0) [16] on normalized expression values. Genes were ranked by variance and used for sample clustering with the ConsensusClusterPlus R package (v1.50.0) [17]. Clustering was conducted using Pearson’s correlation distance, the *k*-means algorithm, and 1’000 bootstrap samples. Each morpho-biotype was then compared against the others in a group-wise manner using DEseq2 R package (v1.26.0) [15]. Differential expression analysis was performed using the Wald test to compute *p*-values for each gene and the Benjamini-Hochberg method [16] to account for multiple hypothesis testing. Genes were deemed significantly differentially expressed if they exhibited an adjusted *p*-value ≤ 0.01 and a linear fold change of at least 2 (either upregulated or downregulated).

CNV estimation

Somatic CNVs for each micro-dissected tumour areas were estimated using the inferCNV R package (v1.10.1) [19]. Using normal pancreas samples derived from GTEX as reference, inferCNV was run with the following parameters --cutoff 0.1 and --noise_filter 0.2. To calculate a CNV value quantifying the overall level of CNVs (both duplications and deletions) in each sample, a CNV scoring method [20] was used. CNVs scores were re-standardized to a mean of zero, scaled from -1 to 1 and then the sum of squared values was calculated as the overall CNV score for each sample.

Enrichment of gene signatures

Transcriptional gene signatures related to biological processes of Synaptic Transmission and Neuronal Differentiation [21] were used to calculate a score

value for each sample. AddModuleScore() function of Seurat R package (v5.0.2) [22] was used with standard parameters except for the number of control features, which was set to the number of genes in the signature when possible.

Motif enrichment analysis

Considering the sequences from -500 bp to +50 bp relative to annotated transcription start sites of differentially expressed genes of the three morpho-biotypes (NCBI Refseq release 200 gene annotations), motif enrichment analysis was performed using Pscan [23] and using a custom set of position-specific weight matrices (PWMs) [24]. PWMs were considered significantly overrepresented when showing a *p*-value ≤ 1e-5.

Analysis of Regulatory Networks

ARACNe-AP [25] was run with 200 bootstrap iterations for the reconstruction of gene regulatory networks using the gene expression matrix based on laser micro-dissection (LMD) data. To identify the relationships among putative regulators and predicted target genes, we used a published list of transcription factors [26] that was further manually curated removing well-known chromatin remodelers and mitochondrial transcriptional regulators (final number of TFs considered was 1’590). ARACNe networks were imported and used by the VIPER (Virtual Inference of Protein Activity by Enriched Regulon analysis) R package (v1.32.0) [27] for the identification of possible master regulators. We compared each morpho-biotype against each other using the msviper algorithm of the VIPER package and the gene signatures previously identified. To infer the enrichment of the top differentially expressed transcription factors, we measured the activity of a regulator based on the enrichment of its target genes in each morpho-biotype. Representative differentially expressed transcription factors and their gene targets were displayed as networks using cytoscape (v.3.8.2) [28].

Single-cell RNAseq data analysis

Dataset Processing

Single-cell RNA-seq data from primary treatment-naive PDACs were obtained from Chan-Seng-Yue et al. [5]. Single-cell data (h5 files) already aligned to hg19 using CellRanger (v.2.1.1) (10x Genomics) were downloaded from EGA archive (EGAC00001000710) and then were imported using the Seurat R toolkit [22]. After the removal of low quality cells (<1’000 detected genes or >25% reads mapping to mitochondrial genome) and of mitochondrial genes from the features, the SCTransform algorithm (v0.4.1) was used to normalize the data [29]. Principal Component Analysis (PCA) was performed and the top PCs (determined as the number of PCs whose percentage of variance increase among consecutive PCs is less than 0.1%) were then used to build a *k*-nearest neighbors’ graph (*k*=20) of single-cell profiles. Community detection using the Leiden clustering method (resolution = 0.8) was performed to identify distinct cell clusters and then different cell populations were identified using known cell type-specific gene markers from [30, 31]. To confirm the presence of clusters enriched for tumour cells, InferCNV (R package; v1.10.1) [19] was used as described above, setting a set of high confidence non-neoplastic cells identified in the same data.

Dataset Integration of scRNAseq data

Harmony (R package; v0.1.1) [32] was used to correct for technical factors between datasets. Using Patient ID as factor, Harmony constructed a well-integrated embedding across cell types and patients. After selecting only tumour cells, batch-corrected harmony PCA components were then used as input for UMAP and downstream clustering approach. A *k*-nearest neighbors' graph (*k*=20) of single tumour cell profiles was built and then Leiden clustering method (resolution = 0.3) was performed to identify distinct tumour cell clusters. Transcriptional signature scores computed by Seurat's AddModulescore() function as described above (see Enrichment of gene signatures) was displayed using Seurat's FeaturePlot() function.

Quality control

The LMD-seq dataset was previously generated [21] from 30 chemotherapy-naive PDAC patients (**Suppl. Table 1**) accurately micro-dissecting a total of 102 tumour samples.

To ensure a high morphological quality from archival formalin-fixed paraffin-embedded (FFPE) human samples, we optimized our RNA-seq pipeline for micro-dissected tumour areas containing approximately 200-500 cells. The sequencing analysis from such samples is inherently challenging due to the tissue processing, which can compromise RNA integrity. The fragmented mRNA can affect gene detection and introduce sequencing artifacts [33]. To mitigate these issues, one of the key optimization steps involved the alignment and counting of sequencing reads on the annotated exons of each mRNA. Initially, we used TopHat2, a standard aligner that relies on genome mapping to identify sequencing positions in the genome potentially originating the read. However, this method showed low performance in quantifying their abundances, with only about 2% to 5% of mapped reads on exons and 1.4% to 2% of unique reads being quantified accurately (**Suppl. Table 2**). Conversely, Kallisto's pseudomapping approach, which employs a statistical framework to account for all the potential transcripts of origin for each sequencing read, significantly enhanced both the accuracy and reliability of gene expression quantification. This method mapped approximately 64% to 76% of the reads on exons and more than doubled the final number of unique reads, achieving 3.6% to 4.7% (**Suppl. Table 2**). Thus, by employing pseudomapping instead of traditional genome mapping, we achieved higher read recovery, enhancing the sensitivity of fragmented RNA quantification from human FFPE laser micro-dissected samples (**Suppl. Table 2**).

The analysis of 102 micro-dissected tumour areas, generated by LMD-seq, unveiled four distinct PDAC cell states (or morpho-biotypes) – Glandular (GL), Transitional (TR), Hybrid (HY), and Undifferentiated (UN) – each characterized by distinct morphological profiles and transcriptional properties [21].

We applied the Similarity Weighted Nonnegative Embedding (SWNE) approach to visually confirm a clear separation of morpho-biotypes in a dimensionally reduced space, and to display the key genes contributing to each group (**Figure 2A**).

The technical quality of LMD-seq was evaluated by examining the coverage distribution for all samples, which determined high quality data distribution across the morpho-biotype annotation for each sample. This result ruled out the possibility of gene expression changes due to technical biases (**Figure 2B**). To verify the robustness of the data, we calculated the number of detected genes

(genes with at least one normalized read count per kb) for each sample across morpho-biotypes. The results were consistent with those obtained from canonical bulk RNA-seq (**Figure 2C**).
The estimate of copy number variation (CNV) levels was calculated for each sample, combining both duplications and deletions. The higher values recorded in the micro-dissected samples compared to normal pancreas indicated the presence of precisely dissected tumour areas, thereby minimizing potential contamination by non-cancer cells during the micro-dissection procedure (**Figure 2D**). Overall, all these analyses confirmed the high accuracy and quality of the generated transcriptomic profiles (**Suppl. Table 2**).

Data Validation

Next, we examined the number of expressed genes across different morpho-biotypes. We specifically focused on highly expressed genes (normalized gene counts per kilobase > 15) and low-expressed genes (normalized gene counts per kilobase < 15), and subsequently calculated the number of expressed genes for each sample (**Figure 3A**). Consistent with the results of the study reporting the main findings related to these datasets [21], tumour areas belonging to the undifferentiated morpho-biotype exhibited a lower number of highly expressed genes and a higher number of lowly expressed genes compared to other samples. The undifferentiated morpho-biotype resembles progenitor-like cells undergoing neuronal lineage priming, expressing low levels of neuronal differentiation and synaptic transmission processes [21]. Enrichment analysis confirmed these signatures were significantly associated only in this morpho-biotype (**Figure 3B** and **Suppl. Table 3**).
Additionally, we compared the performance of our LMD-seq with standard single cell RNAseq (scRNA-seq) in detecting low-expressing genes. To achieve this, we employed publicly available scRNAseq data [5]. After integrating the high-quality CNV-validated single tumour cells from several patients (**Figure 4A**), we performed cell clustering and computed the enrichment scores for neuronal differentiation and synaptic transmission processes across different cell clusters (**Figure 4B-C** and **Suppl. Table 3**). The scRNA-seq data were not able to discriminate tumour cells enriched in non-typically endodermal pathways, such as neuronal differentiation and synaptic transmission. This confirms the superior capability of our LMD-seq approach compared to the commonly used scRNA-seq [21] in identifying rare tumour cells and capturing low-expressed genes.

Reuse potential

Finally, we aimed to identify the transcriptional regulatory networks that sustain the different PDAC morpho-biotypes as one of the potential downstream applications of our dataset. A significant number of transcription factors (TFs) were differentially expressed in each morpho-biotype (**Figure 5A**). These included known endodermal lineage TFs in the glandular morpho-biotype (*e.g.*, SOX9, KLF5, HNF1B, GATA6, ELF3 and FOXA2) [24, 34-37], EMT-associated TFs in the transitional morpho-biotype (*e.g.*, ZEB1 and GLIS2) [38], and TFs related to neural development (POU5F2, POU3F2, NOTO) as well as stemness of basal epithelia (TP63) in the undifferentiated morpho-biotype.
To determine their potential roles in establishing morpho-biotype specific regulatory networks, we first analysed the statistical over-representation of TF

DNA binding motifs in the promoters of differentially expressed genes characteristic of each morpho-biotype (**Figure 5B** and **Suppl. Table 4**). We confirmed the presence of motifs for endodermal lineage TFs such as KLF5, ELF3 and FOXA as enriched in the promoters of genes over-expressed in the glandular morpho-biotype. In addition, motifs bound by the EMT master regulator ZEB1 were also enriched in this group, consistent with ZEB1 acting as a transcriptional repressor [38]. Binding motifs for the EMT activator SNAI2 [39] were instead enriched in the transitional group. On the other hand, promoters of genes over-expressed in the undifferentiated morpho-biotype were enriched for motifs bound by several neuronal lineage TFs such as POU5F2 and other family members, as well as the homeobox TFs NOTO, NKX1-2 and HMX3.
In addition, we sought to identify the transcriptional regulatory networks linking TFs to their target genes across different morpho-biotypes. To achieve this, we first employed a reverse engineering approach to reconstruct the regulatory networks from LMD-seq data, identifying relationships between putative regulators and predicted target genes using ARACNe-AP [25]. This tool quantifies the statistical dependence between expression levels by analysing shared expression patterns across multiple samples, generating a gene-pair association matrix. This matrix is then imported into VIPER algorithm [27] to identify the most active TFs based on the enrichment of their predicted target genes in each morpho-biotype (**Figure 6A** and **Suppl. Table 4**). This analysis not only linked TFs associated with endodermal lineage or EMT programs to their activated and repressed targets but also confirmed the presence of an active neuronal-like network in the undifferentiated morpho-biotype. It suggested possible direct links between neural-enriched TFs and genes associated with functional categories characteristic of the nervous system.

In summary, our data provides a comprehensive reference and resource for understanding the molecular and regulatory mechanisms driving PDAC heterogeneity, highlighted by the identification of novel morpho-biotypes [21]. We further demonstrated that LMD-seq is a valuable spatial transcriptomics approach, capable of robustly capturing histological traits and gene expression patterns. This workflow achieved unprecedented specificity and sensitivity, using as few as ~200 cells, combined with optimized computational analysis. It offers a cost-effective method for conducting discovery studies in several histological-relevant models. To support the exploration and reuse of the LMD-seq dataset, we deployed an interactive data visualization platform [40], enabling researchers and clinicians to intuitively navigate transcriptional profiles of laser micro-dissected PDAC samples, considering individual samples, morpho-biotypes, and genes. This web-based tool enhances transparency, promotes data discovery, and simplifies the integration of LMD-seq datasets into diverse research workflows.

Data Availability

The LMD-seq data, including sequencing raw data, were deposited in the Gene Expression Omnibus (GEO) database. Accession number: GSE209952 (https://www.ncbi.nlm.nih.gov/geo/query/acc.cgi?acc=GSE209952). The scRNA-seq data used in this study are available online at the EGA database (controlled access). A request to access the data can be formulated here

https://ega-archive.org/datasets/EGAD00010001811. The files related to the LMD-seq and single-cell RNA-seq data analysis, including reference files, count matrices, meta data tables and R objects, were deposited on Zenodo at the following repository https://zenodo.org/records/12680172 (DOI: 10.5281/zenodo.12680172). The annotated LMD-seq dataset can be accessed and browsed via an interactive Shiny app (https://pdichiaro.shinyapps.io/lmdseq/), developed using the pre-existing ShinyCell framework (https://github.com/SGDDNB/ShinyCell), while retaining its original license and ensuring compliance with its terms of use. The app provides researchers with a user-friendly platform to explore the data also without any background in computational biology.

Availability of source code and requirements

- Project name: PDAC Morpho-Biotypes
- Project homepage: https://github.com/pdichiaro/PDAC_Morpho-Biotype_Study.
- Operating system(s): Linux operating system
- Programming language: Bash, R
- Other requirements: Anaconda
- License: GNU GPL

The LMD-seq scripts, including processing pipeline, from read trimming to pseudo-mapping, are available online on Github [41], with the corresponding files available on Zenodo [42].
Data analysis R scripts for data visualization and further downstream analysis are also available online on Github [41], with the corresponding files available on Zenodo [42].

Abbreviations

CNV (Copy Number Variation), CDF (Cumulative Distribution Function), FAIR (Findable, Accessible, Interoperable, Reusable), FFPE (Formalin-Fixed Paraffin-Embedded), GL (Glandular), GTEx (Genotype-Tissue Expression project), HY (Hybrid), LMD (Laser Micro-Dissection), NCBI (National Center for Biotechnology Information), PDAC (Pancreatic Ductal Adenocarcinoma), PCA (Principal Component Analysis), PWMs (Position-specific Weight Matrices), RNA-seq (RNA sequencing), TF (Transcription Factor), TR (Transitional), UMAP (Uniform Manifold Approximation and Projection), and UN (Undifferentiated).

Competing interests

The authors declare no potential conflicts of interest.

Author contributions

Conceptualization: PDC, IB
Data curation and analysis: PDC
Supervision: GRD, GN, IB
Funding acquisition: GRD, GN, IB
Writing-original draft: PDC, IB
Writing-review and editing: PDC, GRD, GN, IB

Ethical Approval

This study was approved by the medical ethical committee of the Humanitas Research Hospital IRCCS (Rozzano, Italy).

Consent for Publication

All patients signed informed consent forms for molecular analyses and to the publishing of the data.

Funding

This work was supported by AIRC, the Italian Association for Research on Cancer (AIRC Investigator Grant #27555 to G.N. and AIRC 5x1000 Grants #21147 to GN) and by the Italian Ministry of Health (grant GR-2016-02361721 to G.R.D). I.B. was supported by funding of the Medical University Vienna. This work was also partially supported by the Italian Ministry of Health with the "Ricerca Corrente" and "5x1000" funds to the IEO IRCCS; and with "Ricerca Corrente" and "Ricerca Corrente di Rete" (ACCORD) 2020-2022 funds to Alleanza Contro il Cancro (ACC). P.D.C. was supported by AIRC fellowships for Italy. The single cell RNA-seq datasets from PDAC samples of the PACA-CA cohort used in this research [5] were provided by the Ontario Institute for Cancer Research (O.I.C.R.), Toronto, Ontario, and generated through funding provided by the Government of Ontario.

Acknowledgements

We are grateful to Lucia Nacci and Fabiana Arco for their assistance in data generation and valuable discussions; Francesco Gualdrini for his insights and helpful suggestions on data analysis; Paola Spaggiari and Alessandro Zerbi for providing precious samples. We also thank all the members of the Barozzi and the Natoli labs for discussion and support.

Figure Legends

Figure 1. *Schematic of the optimized Laser Micro-dissection approach (LMD-seq).*
A-B) Diagram of Laser Micro-dissection (LMD) of small tumour areas (**A**) and data analysis (**B**) including pre-processing steps, and downstream applications.

Figure 2. *LMD-seq generates high-depth, high-quality transcriptomic data.*
A) SWNE representation of single tumour areas from LMD-seq, coloured by morpho-biotype. Co-embedded genes (two for each morpho-biotype) are highlighted (dark red).
B) Box plot showing the coverage distribution for all micro-dissected samples, grouped by morpho-biotype.
C) Bar plot showing the number of detected genes for all micro-dissected samples, grouped by morpho-biotype.
D) Box plot showing the CNV score values calculated in tumour PDAC samples (LMD-seq) compared with normal pancreas samples (GTEx). *p*-value calculated using two-sided Wilcoxon rank-sum tests.
GL=Glandular, n=31; TR=Transitional, n=17; HY=Hybrid, n=27; UN=Undifferentiated, n=27.

Figure 3. *LMD-seq captures genes with low levels of expression.*
A-B) Distributions of the number of genes expressed at high level (**A**) or at low level (**B**) across LMD tumour samples, grouped by morpho-biotype.
C) Violin plots showing the enrichment of the indicated biological processes (Synaptic Transmission and Neuronal Differentiation), stratified by morpho-biotype based on LMD-seq data.

Figure 4. *scRNAseq does not detect the low-expressing neuronal genes of undifferentiated morpho-biotype.*
A-B) UMAP of single tumour cells from scRNA-seq data in 13 primary PDACs after dataset integration using Harmony coloured by patient ID (**A**) or by cell clusters (**B**).
C) Violin plots showing the enrichment of the indicated biological processes (Synaptic Transmission and Neuronal Differentiation), stratified by cell clusters based on scRNA-seq data.

Figure 5. *Transcription factors predicted to regulate the morpho-biotypes*.
A) Heatmap showing TFs differentially expressed in PDAC morpho-biotypes. Highly differential TFs (FC $\geq$ 3) and selected relevant TFs are shown. Gene expression modules (#1–8), derived from differentially expressed genes clustered by graph-based clustering approach, are indicated in red on the right. Morpho-Biotype annotations are shown (GL=Glandular; TR=Transitional; HY=Hybrid; UN=Undifferentiated).
B) Motif enrichment analysis at the promoters of differentially expressed genes (from -500 bp to +50 bp relative to the annotated transcription start site). The table shows the PWMs overrepresented in one morpho-biotype relative to the others (p $\leq$ 1E-5), their TF family and their cognate TFs. TFs identified as differentially expressed in different morpho-biotypes are labelled in bold.

Figure 6. *Morpho-Biotype-specific transcriptional regulatory networks*.
Gene regulatory network representation of selected, differentially activated TFs in the three indicated morpho-biotypes, as determined by ARACNe-VIPER [25, 27]. Each node represents a target gene that is predicted to be positively (red) or negatively (blue) regulated by its corresponding TF (TF MoA, mode of action of a TF based on the Spearman's correlation coefficient with expression of its target genes).

Suppl. Table 1. Clinical features of patient cohort included in the study.

Suppl. Table 2. Summary of RNA sequencing alignment results. The table provides in the different sheets the following information: a) Comparisons of sequencing results of ten LMD-seq PDAC samples aligned using either TopHat2 or Kallisto; b) sequencing results of all LMD PDAC samples aligned using Kallisto. Sample annotations and number of expressed genes for each sample are also reported.

Suppl. Table 3. Gene signatures of synaptic transmission and neuronal differentiation. The table provides in the different sheets the following information: a) the list of expressed genes annotated to GO terms related to synaptic transmission processes; b) the list of expressed genes annotated to GO terms related to neuronal differentiation processes; c) the manually curated list of expressed genes related to neuronal differentiation processes; D) a detailed description of the gene signature construction is reported.

Suppl. Table 4. Gene regulatory networks in distinct morpho-biotypes. The table provides in the different sheets the following information: a) the list of all differentially expressed transcription factor genes; b) the position weight matrixes (PWM) used in the motif enrichment analyses; c) the master regulators derived by the VIPER analysis in the different morpho-biotypes. For each PWM the source (database or publication) is indicated while for each master regulator the gene expression levels and log2FC are shown.

References

1. Bengtsson A, Andersson R and Ansari D. The actual 5-year survivors of pancreatic ductal adenocarcinoma based on real-world data. Sci Rep. 2020;10 1:16425. doi:10.1038/s41598-020-73525-y.
2. Verbeke C. Morphological heterogeneity in ductal adenocarcinoma of the pancreas - Does it matter? Pancreatology. 2016;16 3:295-301. doi:10.1016/j.pan.2016.02.004.
3. Jovic D, Liang X, Zeng H, Lin L, Xu F and Luo Y. Single-cell RNA sequencing technologies and applications: A brief overview. Clin Transl Med. 2022;12 3:e694. doi:10.1002/ctm2.694.
4. Juiz N, Elkaoutari A, Bigonnet M, Gayet O, Roques J, Nicolle R, et al. Basal-like and classical cells coexist in pancreatic cancer revealed by single-cell analysis on biopsy-derived pancreatic cancer organoids from the classical subtype. FASEB J. 2020; doi:10.1096/fj.202000363RR.
5. Chan-Seng-Yue M, Kim JC, Wilson GW, Ng K, Figueroa EF, O'Kane GM, et al. Transcription phenotypes of pancreatic cancer are driven by genomic events during tumor evolution. Nat Genet. 2020;52 2:231-40. doi:10.1038/s41588-019-0566-9.
6. Hwang WL, Jagadeesh KA, Guo JA, Hoffman HI, Yadollahpour P, Reeves JW, et al. Single-nucleus and spatial transcriptome profiling of pancreatic cancer identifies multicellular dynamics associated with neoadjuvant treatment. Nat Genet. 2022;54 8:1178-91. doi:10.1038/s41588-022-01134-8.
7. Di Chiaro P, Nacci L, Arco F, Brandini S, Polletti S, Palamidessi A, et al. Mapping functional to morphological variation reveals the basis of regional extracellular matrix subversion and nerve invasion in pancreatic cancer. Cancer Cell. 2024;42 4:662-81 e10. doi:10.1016/j.ccell.2024.02.017.

8. Wilkinson MD, Dumontier M, Aalbersberg IJ, Appleton G, Axton M, Baak A, et al. The FAIR Guiding Principles for scientific data management and stewardship. Sci Data. 2016;3:160018. doi:10.1038/sdata.2016.18.
9. Krueger F: Trim Galore: a wrapper tool around Cutadapt and FastQC to consistently apply quality and adapter trimming to FastQ files, with some extra functionality for MspI-digested RRBS-type (Reduced Representation Bisufite-Seq) libraries. https://www.bioinformatics.babraham.ac.uk/projects/trim_galore/ (2012).
10. Kim D, Pertea G, Trapnell C, Pimentel H, Kelley R and Salzberg SL. TopHat2: accurate alignment of transcriptomes in the presence of insertions, deletions and gene fusions. Genome Biol. 2013;14 4:R36. doi:10.1186/gb-2013-14-4-r36.
11. Bray NL, Pimentel H, Melsted P and Pachter L. Near-optimal probabilistic RNA-seq quantification. Nat Biotechnol. 2016;34 5:525-7. doi:10.1038/nbt.3519.
12. GENCODE Project: Encyclopædia of genes and gene variants. https://www.gencodegenes.org/human/release_33.html. Accessed 01 Oct 2024.
13. Lawrence M, Huber W, Pagès H, Aboyoun P, Carlson M, Gentleman R, et al. Software for computing and annotating genomic ranges. PLoS Comput Biol. 2013;9 8:e1003118. doi:10.1371/journal.pcbi.1003118.
14. Soneson C, Love MI and Robinson MD. Differential analyses for RNA-seq: transcript-level estimates improve gene-level inferences. F1000Res. 2015;4:1521. doi:10.12688/f1000research.7563.2.
15. Love MI, Huber W and Anders S. Moderated estimation of fold change and dispersion for RNA-seq data with DESeq2. Genome Biol. 2014;15 12:550. doi:10.1186/s13059-014-0550-8.
16. Bengtsson H: matrixStats: Methods that Apply to Rows and Columns of Matrices (and to Vectors). https://github.com/HenrikBengtsson/matrixStats. Accessed 14 Nov 2024.
17. Wilkerson MD and Hayes DN. ConsensusClusterPlus: a class discovery tool with confidence assessments and item tracking. Bioinformatics. 2010;26 12:1572-3. doi:10.1093/bioinformatics/btq170.
18. Benjamini Y and Hochberg Y. Controlling the false discovery rate: a practical and powerful approach to multiple testing. Journal of the Royal Statistical Society. 1995;Series B 57:289–300.
19. inferCNV of the Trinity CTAT Project. https://github.com/broadinstitute/inferCNV. Accessed 18 Nov 2024.
20. Peng J, Sun BF, Chen CY, Zhou JY, Chen YS, Chen H, et al. Single-cell RNA-seq highlights intra-tumoral heterogeneity and malignant progression in pancreatic ductal adenocarcinoma. Cell Res. 2019;29 9:725-38. doi:10.1038/s41422-019-0195-y.
21. Di Chiaro P, Nacci L, Arco F, Brandini S, Polletti S, Palamidessi A, et al. Mapping functional to morphological variation reveals the basis of regional extracellular matrix subversion and nerve invasion in pancreatic cancer. Cancer Cell. 2024; doi:10.1016/j.ccell.2024.02.017.
22. Hao Y, Hao S, Andersen-Nissen E, Mauck WM, Zheng S, Butler A, et al. Integrated analysis of multimodal single-cell data. Cell. 2021;184 13:3573-87.e29. doi:10.1016/j.cell.2021.04.048.
23. Zambelli F, Pesole G and Pavesi G. Pscan: finding over-represented transcription factor binding site motifs in sequences from co-regulated or co-expressed genes. Nucleic Acids Res. 2009;37 Web Server issue:W247-52. doi:10.1093/nar/gkp464.
24. Diaferia GR, Balestrieri C, Prosperini E, Nicoli P, Spaggiari P, Zerbi A, et al. Dissection of transcriptional and cis-regulatory control of differentiation in

human pancreatic cancer. EMBO J. 2016;35 6:595-617. doi:10.15252/embj.201592404.

25. Lachmann A, Giorgi FM, Lopez G and Califano A. ARACNe-AP: gene network reverse engineering through adaptive partitioning inference of mutual information. Bioinformatics. 2016;32 14:2233-5. doi:10.1093/bioinformatics/btw216.

26. Lambert SA, Jolma A, Campitelli LF, Das PK, Yin Y, Albu M, et al. The Human Transcription Factors. Cell. 2018;172 4:650-65. doi:10.1016/j.cell.2018.01.029.

27. Alvarez MJ, Shen Y, Giorgi FM, Lachmann A, Ding BB, Ye BH, et al. Functional characterization of somatic mutations in cancer using network-based inference of protein activity. Nat Genet. 2016;48 8:838-47. doi:10.1038/ng.3593.

28. Shannon P, Markiel A, Ozier O, Baliga NS, Wang JT, Ramage D, et al. Cytoscape: a software environment for integrated models of biomolecular interaction networks. Genome research. 2003;13 11:2498-504. doi:10.1101/gr.1239303.

29. Hafemeister C and Satija R. Normalization and variance stabilization of single-cell RNA-seq data using regularized negative binomial regression. Genome Biol. 2019;20 1:296. doi:10.1186/s13059-019-1874-1.

30. Tosti L, Hang Y, Debnath O, Tiesmeyer S, Trefzer T, Steiger K, et al. Single-Nucleus and In Situ RNA-Sequencing Reveal Cell Topographies in the Human Pancreas. Gastroenterology. 2021;160 4:1330-44.e11. doi:10.1053/j.gastro.2020.11.010.

31. Muraro MJ, Dharmadhikari G, Grün D, Groen N, Dielen T, Jansen E, et al. A Single-Cell Transcriptome Atlas of the Human Pancreas. Cell Syst. 2016;3 4:385-94.e3. doi:10.1016/j.cels.2016.09.002.

32. Korsunsky I, Millard N, Fan J, Slowikowski K, Zhang F, Wei K, et al. Fast, sensitive and accurate integration of single-cell data with Harmony. Nat Methods. 2019;16 12:1289-96. doi:10.1038/s41592-019-0619-0.

33. Groelz D, Sobin L, Branton P, Compton C, Wyrich R and Rainen L. Non-formalin fixative versus formalin-fixed tissue: a comparison of histology and RNA quality. Experimental and molecular pathology. 2013;94 1:188-94. doi:10.1016/j.yexmp.2012.07.002.

34. Gao N, LeLay J, Vatamaniuk MZ, Rieck S, Friedman JR and Kaestner KH. Dynamic regulation of Pdx1 enhancers by Foxa1 and Foxa2 is essential for pancreas development. Genes Dev. 2008;22 24:3435-48. doi:10.1101/gad.1752608.

35. Milan M, Balestrieri C, Alfarano G, Polletti S, Prosperini E, Spaggiari P, et al. FOXA2 controls the cis-regulatory networks of pancreatic cancer cells in a differentiation grade-specific manner. EMBO J. 2019;38 20:e102161. doi:10.15252/embj.2019102161.

36. Martinelli P, Carrillo-de Santa Pau E, Cox T, Sainz B, Jr., Dusetti N, Greenhalf W, et al. GATA6 regulates EMT and tumour dissemination, and is a marker of response to adjuvant chemotherapy in pancreatic cancer. Gut. 2017;66 9:1665-76. doi:10.1136/gutjnl-2015-311256.

37. Haumaitre C, Barbacci E, Jenny M, Ott MO, Gradwohl G and Cereghini S. Lack of TCF2/vHNF1 in mice leads to pancreas agenesis. Proc Natl Acad Sci U S A. 2005;102 5:1490-5. doi:10.1073/pnas.0405776102.

38. Balestrieri C, Alfarano G, Milan M, Tosi V, Prosperini E, Nicoli P, et al. Co-optation of Tandem DNA Repeats for the Maintenance of Mesenchymal Identity. Cell. 2018;173 5:1150-64 e14. doi:10.1016/j.cell.2018.03.081.

39. Nieto MA, Sargent MG, Wilkinson DG and Cooke J. Control of cell behavior during vertebrate development by Slug, a zinc finger gene. Science. 1994;264 5160:835-9. doi:10.1126/science.7513443.

40. Di Chiaro P: An interactive Shiny app to explore and visualize LMDseq dataset. https://pdichiaro.shinyapps.io/lmdseq/. Accessed 19 Dec 2024.

41. Di Chiaro P: A repository of code for data analysis/processing generated for the study of PDAC Morpho-Biotypes. https://github.com/pdichiaro/PDAC_Morpho-Biotype_Study. Accessed 27 Aug 2024.
42. Di Chiaro P: Reference files, count matrices, meta data tables and R objects from LMD-seq and single-cell RNA-seq data of PDAC human samples. https://zenodo.org/records/12680172. Accessed 27 Aug 2024.

Figure 1

Click here to access/download;Figure;Fig1.pdf

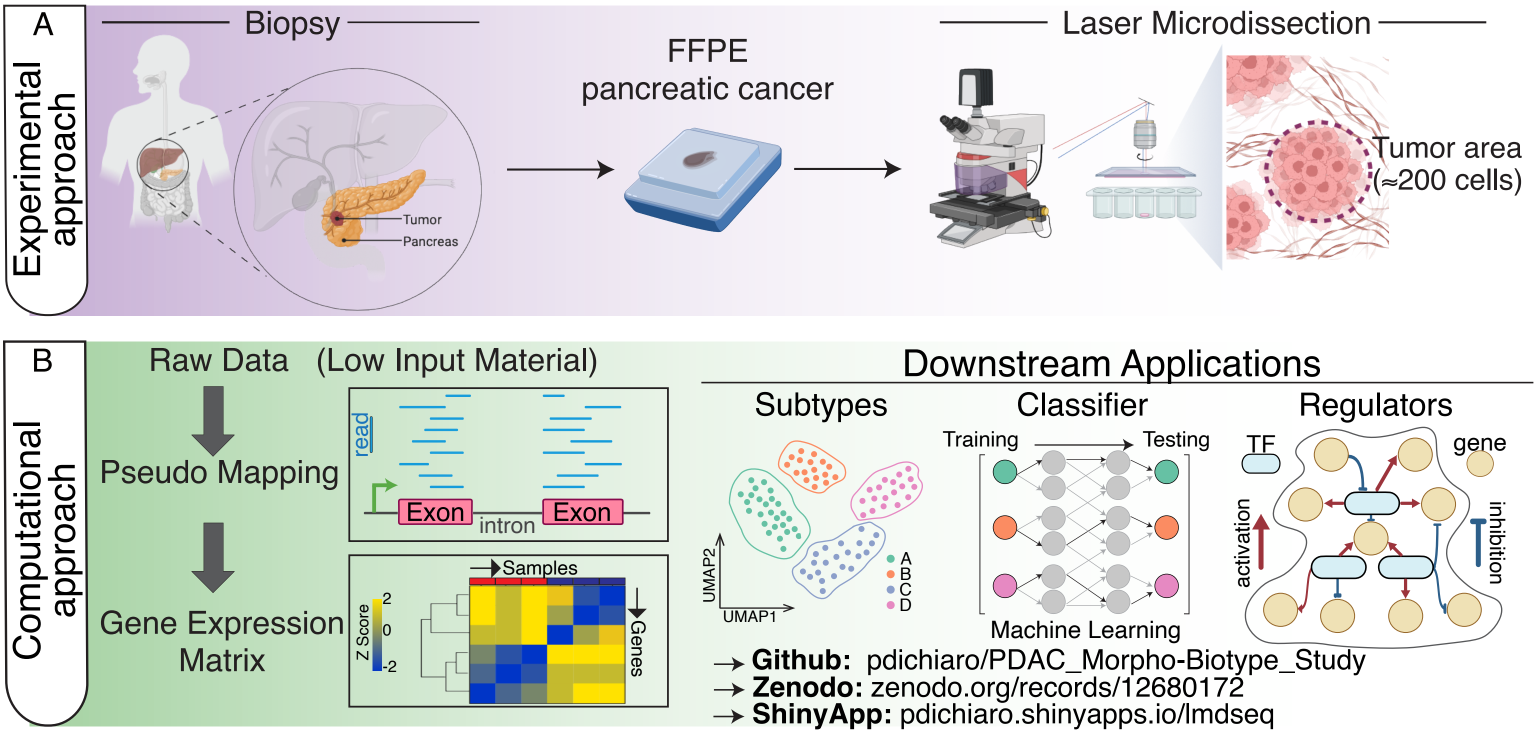

Figure 2

Click here to access/download;Figure;Fig2.pdf

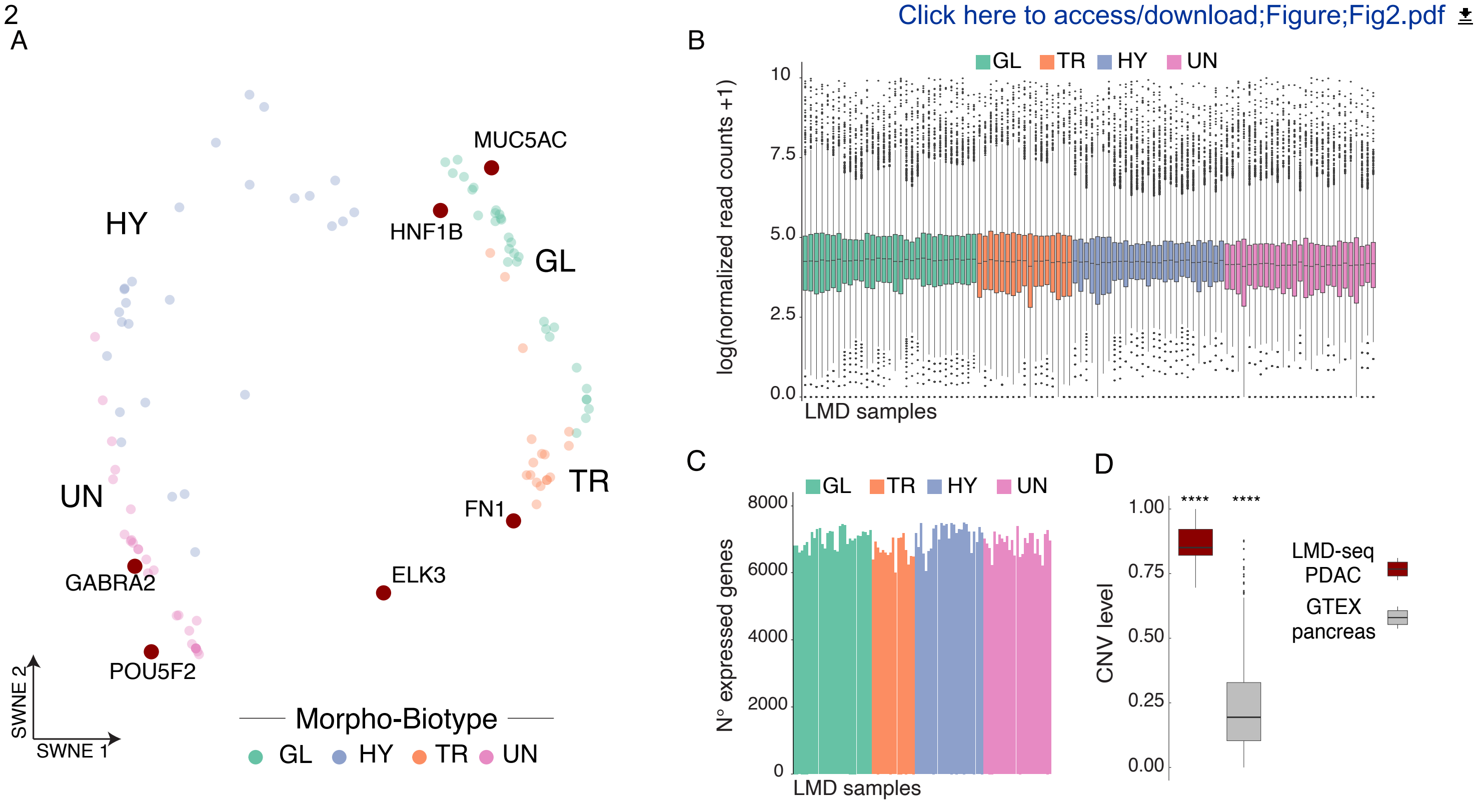

Figure 3

Click here to access/download;Figure;Fig3.pdf

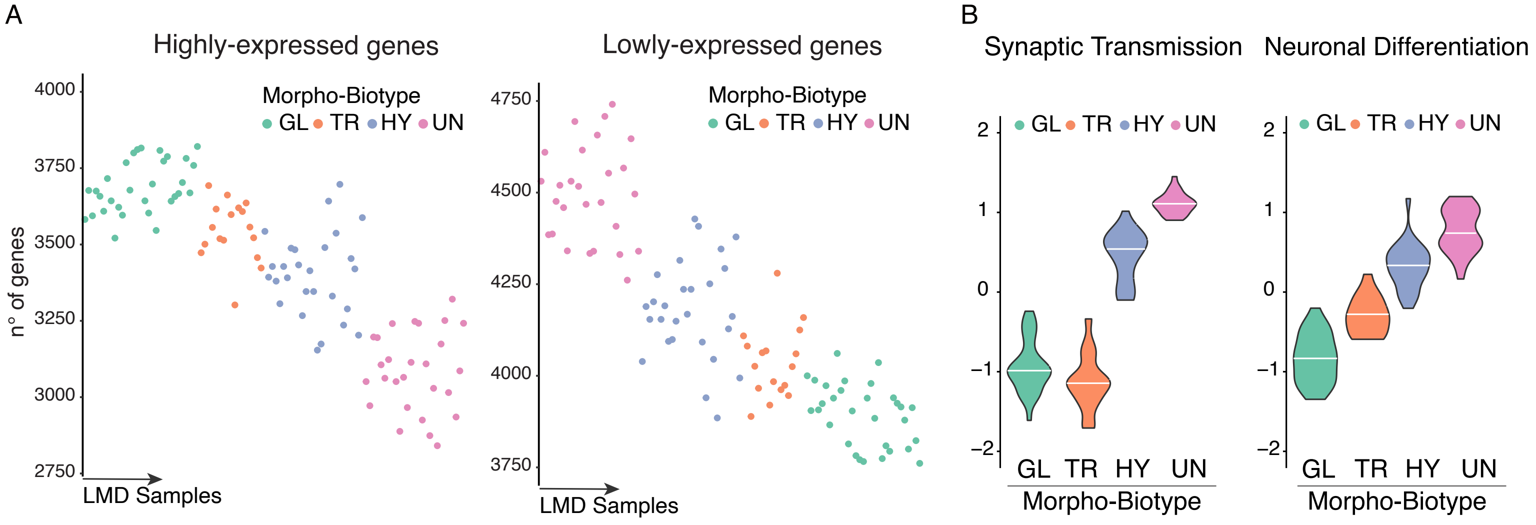

Figure 4

Click here to access/download;Figure;Fig4.pdf

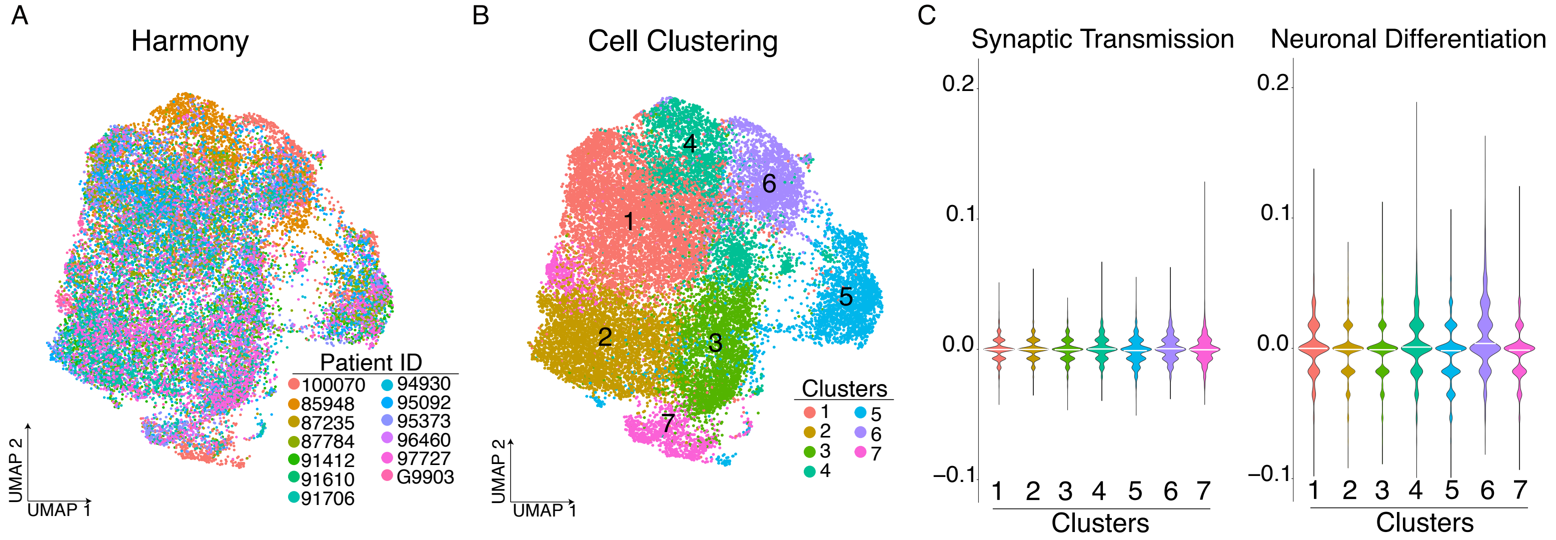

Figure 5

Click here to access/download;Figure;Fig5.pdf

A

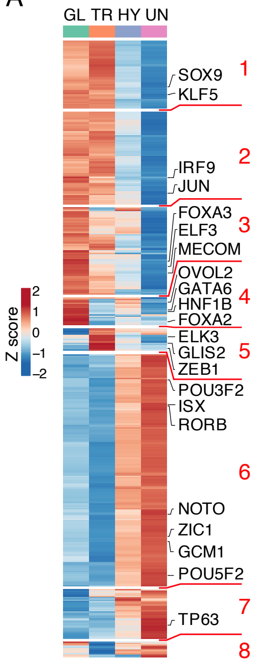

B

PWM	TF FAMILY	p	TF	OTHER TFs	
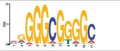	3 ZnF Kruppel TFs	3.66E-24	**KLF5**	**KLF4**, **KLF2**, **EGR1**, **KLF3**, **KLF6**, **SP1**, **KLF13**	Biotype GL
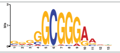	E2F domain	7.25E-22	**E2F4**	**E2F8**, **E2F2**	Biotype GL
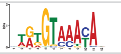	Forkhead box	3.32E-16	**FOXA2**	**FOXA3**, **FOXQ1**	Biotype GL
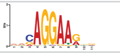	Ets domain	2.78E-16	**ELF3**	**EHF**, **ELF1**, **ETV4**, **ETS2**, **ERF**	Biotype GL
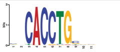	HD-ZF	2.96E-06	**ZEB1**	**ZEB2**	Biotype GL
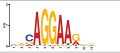	Ets domain	2.04E-07	**ELK3**	**ETS1**, SPI1	Biotype TR
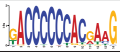	> 3 ZnF TFs	1.49E-06	**SNAI2**	**GLIS2**	Biotype TR
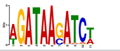	GATA domain	1.23E-33	**GATA3**	-	Biotype UN
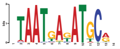	POU domain	1.21E-32	**POU5F2**	**POU5F1B**, **POU3F2**, **POU3F3**, POU2AF1, HDX	Biotype UN
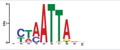	NK domain	2.71E-28	**NOTO**	**NKX1-2**, **MSX1**, **HMX3**, NKX3-1, VAX1, NANOG, VENTX	Biotype UN
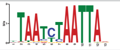	Paired-related HD	4.49E-25	**ISX**	**DMBX1**, **SHOX**, **PRRX1**, **ARGFX**, ALX4, MIXL1	Biotype UN
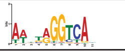	THR-related factors	1.74E-12	**RORB**	**NR1H4**	Biotype UN

Figure 6

Click here to access/download;Figure;Fig6.pdf

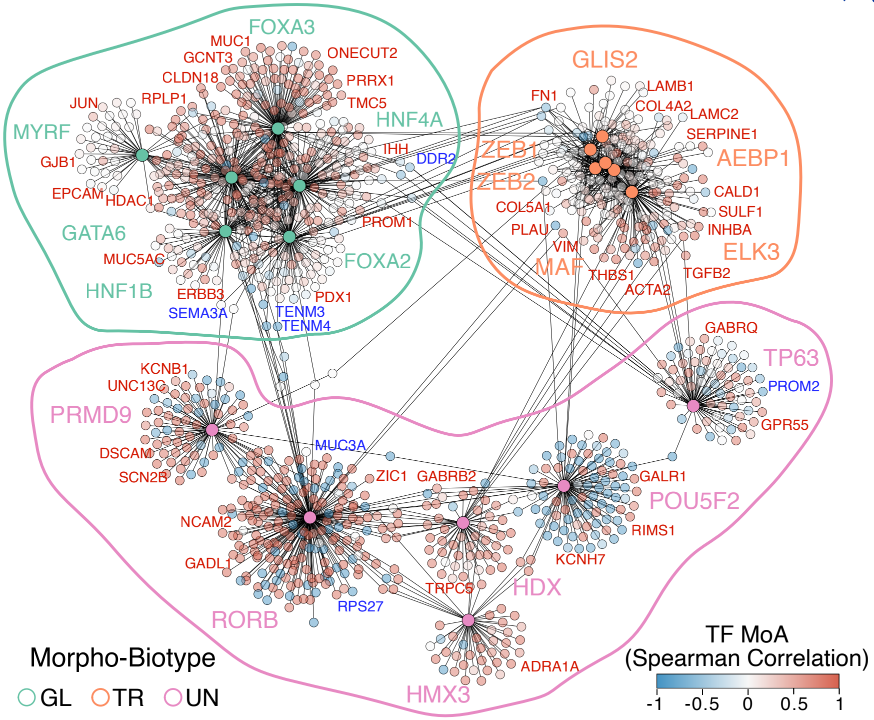

Suppl. Table 1

Click here to access/download
Supplementary Material
Table 1_Supplementary Material.xlsx

Suppl. Table 2

Click here to access/download
Supplementary Material
Table 2_Supplementary Material.xlsx

Suppl. Table 3

Click here to access/download
Supplementary Material
Table 3_Supplementary Material.xlsx

Suppl. Table 4

Click here to access/download
Supplementary Material
Table 4_Supplementary Material.xlsx

Cover letter
Click here to access/download;Personal Cover;Cover Letter.docx

MEDICAL UNIVERSITY OF VIENNA

Center for Cancer Research
Comprehensive Cancer Center,
Medical University of Vienna,
Borschkegasse 8a,
1090 Vienna (Austria)

IEO
European Institute of Oncology

Department of Experimental Oncology
Via Adamello 16,
20139 Milan (Italy)

December 19, 2024

Dear Editors,

On behalf of all co-authors, we are pleased to submit our manuscript entitled "**A framework to mine laser microdissection-based omics data and uncover regulators of pancreatic cancer heterogeneity**" for consideration as Data Note in *GigaScience*.

Following our previous conversation with Dr. Hans Zauner, we appreciated your insights and the willingness to consider this manuscript for review. Based on our correspondence, we are now submitting a novel version of the manuscript that further highlight the added value of this work compared to the original observations reported in *Di Chiaro et al., Cancer Cell, 2024.*

Pancreatic ductal adenocarcinoma (PDAC) is characterized by profound intratumor heterogeneity, which complicates transcriptomic analyses and limits the ability of standard RNA-seq and bioinformatics pipelines to fully capture the underlying complexity. Building on the raw data and observations from our previous study, this manuscript provides a significant enhancement by offering a comprehensive framework for both mining and interpreting the data, and that could be extended to further datasets. This framework not only improves and validates the reproducibility and accessibility of the data, but also introduces novel insights into the regulators of PDAC morpho-biotypes, providing a deeper understanding of the molecular mechanisms underlying tumor heterogeneity. More specifically, our manuscript provides:

- **Methodological and analytical details** for the optimized RNA sequencing of laser microdissected (LMD) samples, enabling researchers to achieve higher coverage and accuracy in gene expression quantification compared to standard pipelines.
- **Demonstration of superior sensitivity** in detecting lowly expressed genes, as compared to single-cell RNA-seq, particularly in the identification of rare tumor cell populations and transcriptional programs with low expression.
- A **new, detailed map of predicted regulatory networks** underlying distinct PDAC morpho-biotypes, which provide novel insights into regulatory mechanisms and key

regulators associated with tumor heterogeneity, demonstrating one novel application of this type of dataset for the community.

- Fully documented **code and processed data objects**, including computational predictions of regulator activities, to facilitate data reuse, reproducibility, and community-driven exploration.
- An **interactive ShinyApp** for user-friendly data visualization, enabling intuitive exploration of the LMD-seq dataset. This tool enhances transparency, promotes discovery, and simplifies integration into diverse research workflows.

In summary, we believe this manuscript represents a valuable resource for the research community, enabling both experimental researchers and computational biologists to better investigate the transcriptional complexity of PDAC and other cancers. By addressing the limitations of standard methodologies, enhancing data accessibility, and integrating regulatory network predictions, we believe this work aligns closely with the aims of *GigaScience*.

Thank you for considering this manuscript for publication. Please do not hesitate to contact us if you require further information.

Thank you for your time and consideration.

Sincerely,

Iros Barozzi, PhD

Pierluigi Di Chiaro, PhD
