# Supplementary material for: A framework to mine laser microdissection-based omics data and uncover regulators of pancreatic cancer heterogeneity
Source: Gigascience. 2025 Sep 5;14:giaf101. doi: 10.1093/gigascience/giaf101 (PMC12412123; doi:10.1093/gigascience/giaf101)
Supplement: giaf101_Authors_Response_To_Reviewer_Comments_Revision_1 [file giaf101_authors_response_to_reviewer_comments_revision_1.pdf]

Author's Response To Reviewer Comments

Point_by_point_reply was added as supplementary material. Also copy-pasted below.

Reviewer #1:
The authors have substantially revised their manuscript and have addressed the majority of my original concerns. I appreciate the addition of new analyses, clarifications regarding alignment tools, improvements in the regulatory network validation, and overall enhancements to methodological transparency. The revised manuscript now presents a more reproducible and comprehensive framework for investigating intra-tumor heterogeneity in PDAC using laser microdissection-based omics.
However, a few important issues remain insufficiently addressed:

REVIEWER QUESTION:
Major Concerns
1. The revised manuscript confirms the use of k-means clustering in combination with Pearson's correlation distance. This approach is statistically problematic, as k-means assumes Euclidean space and spherical clusters, assumptions violated when using correlation-based distances. While the authors reference prior work using similar methods, no justification is provided for this choice nor any sensitivity analysis with more appropriate clustering techniques (e.g., hierarchical clustering or PAM). This remains a methodological flaw that may affect the stability and interpretability of the identified morpho-biotypes.

RESPONSE:
We thank the reviewer for raising this important methodological point. In response, we re-ran the clustering analysis using hierarchical clustering (with Euclidean distance, on scaled gene expression values, and the ward.D2 linkage method). Using this alternative method, we were able to obtain four large, quite pure clusters, enriched for morpho-biotype classification (and an additional smaller cluster). We have incorporated these new results into the manuscript and added a detailed description in the Methods section to address this point.

REVIEWER QUESTION:
2. The manuscript continues to use AddModuleScore(), a function developed for single-cell expression matrices to estimate gene signature enrichment in bulk RNA-seq data. Bulk data represent population-level averages and do not preserve cell-level variability required for AddModuleScore's binning and control gene selection. This use may lead to misleading interpretations of pathway enrichment. The authors should have either used bulk-appropriate methods (e.g., GSVA or ssGSEA) or included a justification and discussion of this limitation.

RESPONSE:
We thank the reviewer for this comment. We have performed an additional analysis using two widely used approached from the GSVA R package: gene set variation analysis (GSVA) and single-sample gene set enrichment analysis (ssGSEA). We added them in the main manuscript (Figure 3D in the revised version of the manuscript). As described in the previous point-to-point response (which already included this data) the results are highly consistent with those obtained with the AddModuleScore function.

REVIEWER QUESTION:
Minor
1. While the authors now include a pseudobulk reference from endocrine cells derived from scRNA-seq data, GTEx samples remain a primary comparator for CNV estimation. The inherent heterogeneity and technical differences in GTEx processing introduce potential biases. This limitation is acknowledged in the revised manuscript, and the additional pseudobulk reference helps mitigate the concern, though patient-matched normals would have been ideal.

RESPONSE:
We agree with the reviewer that patient-matched normal tissues are ideal for accurate CNV inference, but, unfortunately we do not have access to this kind of samples in our study. As mentioned in the previous

point-to-point response, we already added a confirmation analysis, in which we re-ran inferCNV with the same parameters but using a pseudobulk reference form normal endocrine cells. The CNV patterns derived from this analysis showed extreme consistency with those detected from the LMD-seq microdissected tumor samples using the GTEx as reference, reinforcing the robustness of the results we previously obtained (Figure 2D).

REVIEWER QUESTION:
2. The manuscript continues to use a fixed threshold of 15 normalized counts per kilobase to define highly vs. lowly expressed genes. Although the authors acknowledge the arbitrariness of this cutoff, no further validation, sensitivity analysis, or alternative statistical criteria are provided. This limits the generalizability and reproducibility of the expression stratification.

RESPONSE:
To answer to this doubt we, we have now generated an additional analysis calculating the number of expressed genes using two additional thresholds. This yielded results consistent with those obtained using the threshold of 15 normalized counts per kilobase (Figure 3B in the revised version of the manuscript).

REVIEWER COMMENT:
Summary
Overall, this is a well-structured pipeline for LMD-based spatial transcriptomics in PDAC. Nonetheless, two critical methodological issues remain; the clustering strategy and gene signature scoring method which warrant careful reconsideration or, at a minimum, explicit acknowledgment of their limitations.

RESPONSE:
We thank the reviewer for recognizing the quality and structure of our pipeline for LMD-based spatial transcriptomics, as well as its potential applicability to other models. We acknowledge the two key methodological concerns, which are now carefully and fully addressed in the revised version of the manuscript.

Editorial Manager®

Cookie Preference Center

We use cookies which are necessary to make our site work. We may also use additional cookies to analyze, improve and personalize our content and your digital experience. For more information, see our Cookie Policy.

You may choose not to allow some types of cookies. However, blocking some types may impact your experience of our site and the services we are able to offer. See the different category headings below to find out more or change your settings.

Allow all

Manage Consent Preferences

Strictly Necessary Cookies

Always active

These cookies are necessary for the website to function and cannot be switched off in our systems. They are usually only set in response to actions made by you which amount to a request for services, such as setting your privacy preferences, logging in or filling in forms. You can set your browser to block or alert you about these cookies, but some parts of the site will not then work.

Functional Cookies

These cookies enable the website to provide enhanced functionality and personalisation. They may be set by us or by third party providers whose services we have added to our pages. If you do not allow these cookies then some or all of these services may not function properly.

Performance Cookies

These cookies allow us to count visits and traffic sources so we can measure and improve the performance of our site. They help us to know which pages are the most and least popular and see how visitors move around the site.

Targeting Cookies

These cookies may be set through our site by our advertising partners. They may be used by those companies to build a profile of your interests and show you relevant adverts on other sites. If you do not allow these cookies, you will experience less targeted advertising.

Cookie List

Clear

Apply Cancel

Consent Leg.Interest

Confirm my choices

Powered by onetrust
