# Supplementary material for: A framework to mine laser microdissection-based omics data and uncover regulators of pancreatic cancer heterogeneity
Source: Gigascience. 2025 Sep 5;14:giaf101. doi: 10.1093/gigascience/giaf101 (PMC12412123; doi:10.1093/gigascience/giaf101)
Supplement: giaf101_Reviewer_1_Report_Original_Submission — Thazin Aung -- 2/2/2025 [file giaf101_reviewer_1_report_original_submission.pdf]

**Reviewer Report**

**Title: A framework to mine laser microdissection-based omics data and uncover regulators of pancreatic cancer heterogeneity**

**Version: Original Submission** **Date:** 2/2/2025

**Reviewer name: Thazin Aung**

**Reviewer Comments to Author:**

In summary, this study focused on pancreatic ductal adenocarcinoma (PDAC) and its intra-tumor heterogeneity. The researchers developed and validated LMD-seq, an optimized RNA sequencing method for spatially resolved laser micro-dissected tumor areas. The authors reported that this method improves detection of lowly-expressed genes compared to single-cell RNA-seq, particularly identifying rare tumor cell populations and transcriptional programs. They mapped regulatory networks linked to distinct PDAC morpho-biotypes, uncovering novel mechanisms and key regulators. Their work provides reproducible workflows, datasets, and computational tools for studying PDAC heterogeneity and potential applications in other cancers.The study presents a technically innovative and promising approach for analyzing pancreatic ductal adenocarcinoma (PDAC) heterogeneity using LMD-seq. However, several methodological flaws and inconsistencies need to be addressed to enhance the study's credibility and impact.Major comments1. TopHat2 (v2.1.1) is outdated and has been replaced by HISAT2 or STAR, which offer better accuracy and efficiency in RNA-seq alignment. Using TopHat2 may reduce sensitivity in splice junction detection, affecting transcriptome analysis. Additionally, Kallisto (v0.46.0) is a pseudo-aligner and does not perform traditional genome alignment like TopHat2. The study should clarify why both approaches are used and how their results are integrated for analysis.2. K-means clustering assumes Euclidean distance and works best with spherical clusters in continuous space. However, the study uses Pearson's correlation distance, which measures similarity in expression patterns but does not preserve the Euclidean space required for k-means.Using Pearson's correlation distance with k-means violates its assumptions and may lead to incorrect or unstable clustering results.3. Using bulk RNA-seq from GTEx as a reference may introduce biases because normal pancreas tissue contains multiple cell types, leading to heterogeneous expression profiles that may not correctly reflect a normal diploid state. GTEx samples are processed differently (different protocols, sequencing depths, etc.), which can introduce technical artifacts instead of real CNVs. The lack of patient-matched normal samples can lead to incorrect CNV inference due to inter-individual variability.4. Seurat's AddModuleScore() function relies on per-cell expression data, calculating gene set enrichment at the single-cell level. Using this function for bulk RNA-seq data may produce misleading scores since bulk RNA-seq represents an average expression across cell populations rather than individual cells.5. For Single-cell RNAseq data processing, the study uses single-cell RNA-seq data aligned to hg19, an older human genome reference, while their bulk RNA-seq analysis (LMD-seq) is mapped to hg38 (GRCh38). This inconsistency can lead to mapping biases, gene annotation mismatches, and difficulties in integrating bulk and single-cell data. hg38 includes more accurate gene models, better representation of repetitive regions, and improved transcript annotation compared to hg19.6. The study incorrectly

justifies using Kallisto over TopHat2 for FFPE RNA-seq by comparing their performance. TopHat2 is outdated and poorly suited for FFPE samples due to its inefficient spliced-read detection. However, comparing TopHat2 (a traditional genome aligner) with Kallisto (a pseudomapper) is flawed because Kallisto does not perform actual genome alignment but rather estimates transcript abundances probabilistically. The reported mapping percentages (2-5% for TopHat2 vs. 64-76% for Kallisto) are not directly comparable since they measure different aspects of read assignment. A better approach would be using modern genome aligners like HISAT2 or STAR instead of TopHat2 for a fair comparison.7. The study incorrectly associates ZEB1 with the glandular morpho-biotype, despite its well-known role in EMT and dedifferentiation, making this classification biologically inconsistent. ZEB1 expression levels should be re-evaluated, and experimental validation (e.g., ChIP-seq, ATAC-seq) should confirm its actual regulatory activity.Mior Comments1. Figure 2: Misinterpretation of CNV ScoresComparing CNV scores between LMD-seq PDAC samples and GTEx normal pancreas is problematic because CNV estimation is designed for tumor samples and applying it to normal tissues may introduce artificial differences that do not reflect true tumor-specific CNVs.2. Figure 3: Lack of Clarity in Gene Expression CategoriesThe definitions of "highly expressed genes" (>15 normalized gene counts per kilobase) and "lowly expressed genes" lack justification or references, making the threshold selection arbitrary and potentially non-reproducible.3. Figure 5: Misclassification of ZEB1's RoleThe legend suggests ZEB1 functions as a repressor in the glandular morpho-biotype, but ZEB1 is an EMT-promoting factor typically linked to dedifferentiation, making its classification in the glandular subtype biologically inconsistent.4. Figure 6: Misinterpretation of Regulatory NetworksThe regulatory network analysis does not explain how inferred TF-target relationships were validated. Since ARACNe-VIPER relies on statistical dependencies rather than direct binding data, experimental validation (e.g., ChIP-seq) is needed to confirm biological relevance.

**Level of Interest**

Please indicate how interesting you found the manuscript: Choose an item.

**Quality of Written English**

Please indicate the quality of language in the manuscript: Choose an item.

**Declaration of Competing Interests**

Please complete a declaration of competing interests, considering the following questions:

- Have you in the past five years received reimbursements, fees, funding, or salary from an organisation that may in any way gain or lose financially from the publication of this manuscript, either now or in the future?
- Do you hold any stocks or shares in an organisation that may in any way gain or lose financially from the publication of this manuscript, either now or in the future?

- Do you hold or are you currently applying for any patents relating to the content of the manuscript?
- Have you received reimbursements, fees, funding, or salary from an organization that holds or has applied for patents relating to the content of the manuscript?
- Do you have any other financial competing interests?
- Do you have any non-financial competing interests in relation to this paper?

If you can answer no to all of the above, write 'I declare that I have no competing interests' below. If your reply is yes to any, please give details below.

I declare that I have no competing interests

I agree to the open peer review policy of the journal. I understand that my name will be included on my report to the authors and, if the manuscript is accepted for publication, my named report including any attachments I upload will be posted on the website along with the authors' responses. I agree for my report to be made available under an Open Access Creative Commons CC-BY license (http://creativecommons.org/licenses/by/4.0/). I understand that any comments which I do not wish to be included in my named report can be included as confidential comments to the editors, which will not be published.

Choose an item.

To further support our reviewers, we have joined with Publons, where you can gain additional credit to further highlight your hard work (see: https://publons.com/journal/530/gigascience). On publication of this paper, your review will be automatically added to Publons, you can then choose whether or not to claim your Publons credit. I understand this statement.

Yes Choose an item.
