# Supplementary material for: A framework to mine laser microdissection-based omics data and uncover regulators of pancreatic cancer heterogeneity
Source: Gigascience. 2025 Sep 5;14:giaf101. doi: 10.1093/gigascience/giaf101 (PMC12412123; doi:10.1093/gigascience/giaf101)
Supplement: giaf101_Reviewer_1_Report_Revision_1 — Thazin Aung -- 6/10/2025 [file giaf101_reviewer_1_report_revision_1.pdf]

Reviewer Report

Title: A framework to mine laser microdissection-based omics data and uncover regulators of pancreatic cancer heterogeneity

Version: Revision 1 **Date:** 6/10/2025

Reviewer name: Thazin Aung

Reviewer Comments to Author:

The authors have substantially revised their manuscript and have addressed the majority of my original concerns. I appreciate the addition of new analyses, clarifications regarding alignment tools, improvements in the regulatory network validation, and overall enhancements to methodological transparency. The revised manuscript now presents a more reproducible and comprehensive framework for investigating intra-tumor heterogeneity in PDAC using laser microdissection-based omics.However, a few important issues remain insufficiently addressed:Major ConcernsThe revised manuscript confirms the use of k-means clustering in combination with Pearson's correlation distance. This approach is statistically problematic, as k-means assumes Euclidean space and spherical clusters, assumptions violated when using correlation-based distances. While the authors reference prior work using similar methods, no justification is provided for this choice nor any sensitivity analysis with more appropriate clustering techniques (e.g., hierarchical clustering or PAM). This remains a methodological flaw that may affect the stability and interpretability of the identified morpho-biotypes.The manuscript continues to use AddModuleScore(), a function developed for single-cell expression matrices to estimate gene signature enrichment in bulk RNA-seq data. Bulk data represent population-level averages and do not preserve cell-level variability required for AddModuleScore's binning and control gene selection. This use may lead to misleading interpretations of pathway enrichment. The authors should have either used bulk-appropriate methods (e.g., GSVA or ssGSEA) or included a justification and discussion of this limitation.MinorWhile the authors now include a pseudobulk reference from endocrine cells derived from scRNA-seq data, GTEx samples remain a primary comparator for CNV estimation. The inherent heterogeneity and technical differences in GTEx processing introduce potential biases. This limitation is acknowledged in the revised manuscript, and the additional pseudobulk reference helps mitigate the concern, though patient-matched normals would have been ideal.The manuscript continues to use a fixed threshold of 15 normalized counts per kilobase to define highly vs. lowly expressed genes. Although the authors acknowledge the arbitrariness of this cutoff, no further validation, sensitivity analysis, or alternative statistical criteria are provided. This limits the generalizability and reproducibility of the expression stratification.SummaryOverall, this is a well-structured pipeline for LMD-based spatial transcriptomics in PDAC. Nonetheless, two critical methodological issues remain; the clustering strategy and gene signature scoring method which warrant careful reconsideration or, at a minimum, explicit acknowledgment of their limitations.

Level of Interest

Please indicate how interesting you found the manuscript: Choose an item.

Quality of Written English

Please indicate the quality of language in the manuscript: Choose an item.

Declaration of Competing Interests

Please complete a declaration of competing interests, considering the following questions:

- Have you in the past five years received reimbursements, fees, funding, or salary from an organisation that may in any way gain or lose financially from the publication of this manuscript, either now or in the future?
- Do you hold any stocks or shares in an organisation that may in any way gain or lose financially from the publication of this manuscript, either now or in the future?
- Do you hold or are you currently applying for any patents relating to the content of the manuscript?
- Have you received reimbursements, fees, funding, or salary from an organization that holds or has applied for patents relating to the content of the manuscript?
- Do you have any other financial competing interests?
- Do you have any non-financial competing interests in relation to this paper?

If you can answer no to all of the above, write 'I declare that I have no competing interests' below. If your reply is yes to any, please give details below.

I declare that I have no competing interests

I agree to the open peer review policy of the journal. I understand that my name will be included on my report to the authors and, if the manuscript is accepted for publication, my named report including any attachments I upload will be posted on the website along with the authors' responses. I agree for my report to be made available under an Open Access Creative Commons CC-BY license (http://creativecommons.org/licenses/by/4.0/). I understand that any comments which I do not wish to be included in my named report can be included as confidential comments to the editors, which will not be published.

Choose an item.

To further support our reviewers, we have joined with Publons, where you can gain additional credit to further highlight your hard work (see: https://publons.com/journal/530/gigascience). On publication of this paper, your review will be automatically added to Publons, you can then choose whether or not to claim your Publons credit. I understand this statement.

Yes Choose an item.
