# Supplementary material for: A framework to mine laser microdissection-based omics data and uncover regulators of pancreatic cancer heterogeneity
Source: Gigascience. 2025 Sep 5;14:giaf101. doi: 10.1093/gigascience/giaf101 (PMC12412123; doi:10.1093/gigascience/giaf101)
Supplement: giaf101_Reviewer_2_Report_Original_Submission — Qingnan Liang, Ph.D. -- 3/21/2025 [file giaf101_reviewer_2_report_original_submission.pdf]

Reviewer Report

Title: A framework to mine laser microdissection-based omics data and uncover regulators of pancreatic cancer heterogeneity

Version: Original Submission **Date:** 3/21/2025

Reviewer name: Qingnan Liang, Ph.D.

Reviewer Comments to Author:

The authors report a framework of incorporating laser micro-dissection in sample collection for RNA-seq, that aims at preserving the spatial information to some extent. The authors suggest that the LMD-seq data has higher quality than single-cell RNA-seq in terms of rare cell population identification and lowly-expressed gene detection. Although there are approaches emerging from laser-capture related experiments that target spatial-genomics, the data quality introduced in this study is far from satisfactory. For example, no spatial analysis could be done with the data, and 200-500 cells are too many for studying fine structure/population of tumor and TME. I feel that the scope might shift to more of the diagnosis area, that sampling a few tumor cores and learn their CNV profiles (would need to use DNA-seq), and maybe compare them across patients (I mean, DNA-seq), instead of doing many downstream analysis with limited overall sample size. Besides, the writing lack many information to judge the analysis and quality of this research. I would recommend rejection.Other comments:1. Do the authors think the LMD captured normal regions are better controls for running inferCNV?2. What would be the best strategy for doing LMD-seq when tumor cores are large and with immune infiltration?3. The comparison between LMD-seq and RNA-seq are not well calibrated: the comparison need to include the sequencing depth.4. Figure 4 is not done seriously. Let alone whether we should integrate tumor scRNA-seq data from different patients, the authors should not plot scaled gene expression values.

Level of Interest

Please indicate how interesting you found the manuscript: Choose an item.

Quality of Written English

Please indicate the quality of language in the manuscript: Choose an item.

Declaration of Competing Interests

Please complete a declaration of competing interests, considering the following questions:

- Have you in the past five years received reimbursements, fees, funding, or salary from an organisation that may in any way gain or lose financially from the publication of this manuscript, either now or in the future?

- Do you hold any stocks or shares in an organisation that may in any way gain or lose financially from the publication of this manuscript, either now or in the future?
- Do you hold or are you currently applying for any patents relating to the content of the manuscript?
- Have you received reimbursements, fees, funding, or salary from an organization that holds or has applied for patents relating to the content of the manuscript?
- Do you have any other financial competing interests?
- Do you have any non-financial competing interests in relation to this paper?

If you can answer no to all of the above, write 'I declare that I have no competing interests' below. If your reply is yes to any, please give details below.

I declare that I have no competing interests

I agree to the open peer review policy of the journal. I understand that my name will be included on my report to the authors and, if the manuscript is accepted for publication, my named report including any attachments I upload will be posted on the website along with the authors' responses. I agree for my report to be made available under an Open Access Creative Commons CC-BY license (http://creativecommons.org/licenses/by/4.0/). I understand that any comments which I do not wish to be included in my named report can be included as confidential comments to the editors, which will not be published.

Choose an item.

To further support our reviewers, we have joined with Publons, where you can gain additional credit to further highlight your hard work (see: https://publons.com/journal/530/gigascience). On publication of this paper, your review will be automatically added to Publons, you can then choose whether or not to claim your Publons credit. I understand this statement.

Yes Choose an item.
